# Investigating Polyethylene Solubility for Solvent-Based Recycling: Experiments and SAFT‑γ Mie Predictions

**DOI:** 10.1021/acs.macromol.6c00489

**Published:** 2026-06-08

**Authors:** Riccardo Standish, Jian Yin, Jakob Burger, George Jackson, Claire S. Adjiman, Mirjana Minceva, Amparo Galindo

**Affiliations:** † Department of Chemical Engineering, Sargent Centre for Process Systems Engineering, 4615 Imperial College London, London SW7 2AZ, U.K.; ‡ TUM School of Life Sciences, 9184 Technical University of Munich, Freising, 85354, Germany; § Campus Straubing for Biotechnology and Sustainability, Technical University of Munich, Straubing, 94315, Germany

## Abstract

Solvent-based dissolution/precipitation plastic recycling processes are emerging as a promising alternative to mechanical recycling, enabling the selective dissolution of polymers from contaminated plastic waste streams. Identifying suitable solvents for such processes requires an accurate assessment of polymer solubility under practical conditions; this is currently possible for very few solvent/polymer combinations. Thermodynamic modeling using molecular-scale models such as the SAFT-γ Mie group-contribution (GC) approach can significantly reduce reliance on experiments that are often time-consuming and costly, and may be integrated into process design workflows. However, polymer solubility data remain scarce, and the modeling of polymer + solvent mixtures poses several challenges, including variations in polymer melting properties and polydispersity. In this study, we present an extensive set of solubility data for four polyethylene (PE) samples, differing in molecular weight, density, and melting properties, in ten solvents and at multiple temperatures, obtained using a polythermal method. We assess the performance of SAFT-γ Mie in predicting the solubility of PE in eight solvents. A very good overall agreement is observed for both solid–liquid and solid–liquid–liquid equilibria; these results confirm the transferability of previously published parameters to complex polymer systems. Experimental and modeling results indicate that the highest solubility at a given temperature is achieved using decalin, a bicyclic solvent, and the aromatic solvents toluene, *p*-xylene, and mesitylene. The solubility of PE in several bioderived solvents, such as limonene, *p*-cymene, and α-pinene, is found to be comparable to that in conventional aromatic solvents, suggesting their suitability as environmentally benign alternatives. Furthermore, our findings indicate that the polymer melting properties have a more significant influence on PE solubility than polymer molecular weight. In this work we provide critical data and model validation, paving the way for the future integration of the SAFT-γ Mie GC approach in computer-aided molecular and process design for solvent-based dissolution/precipitation plastic recycling.

## Graphical abstract

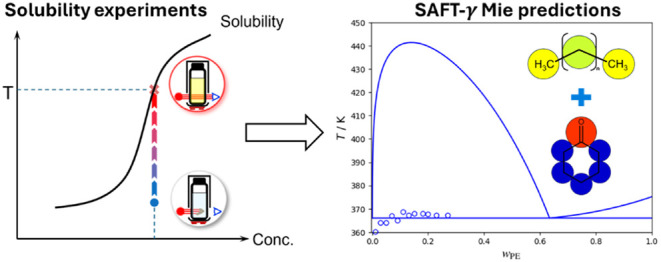

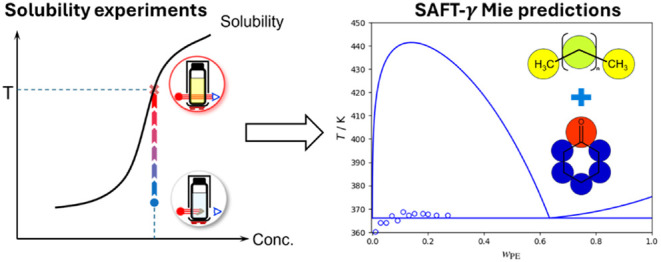

## Introduction

Polyethylene (PE), a synthetic polymeric material whose first industrial production dates back almost a century, remains the most widely manufactured plastic today.^1^ The reason for its success, particularly in packaging and consumer goods, is largely attributed to its durability, chemical resistance, and lightweight nature. However, as societal awareness of the need for more sustainable production and consumption grows, these very features, while beneficial in function, pose significant challenges and are driving a shift toward a circular economy in which reuse and minimally destructive recycling have an important role to play.^2^

Mechanical recycling is currently the most common method for recycling thermoplastics.^3^ It is a physical process in which waste plastics are melted and reprocessed into new materials after being sorted, washed, and shredded into the required form. During the process, the chemical identity of the polymer remains unaltered as it is not fragmented into its constituent monomers. While cost-effective and scalable, this approach faces challenges relating to the presence of impurities or contaminants from waste streams and unforeseen degradation of the polymer, e.g., formation of aldehydes and hydrocarbons induced by heating and oxidation.^4^ These undesired effects result in reduced material properties and product downgrading.^5^

Chemical recycling, on the other hand, involves a chemical decomposition of plastics into their monomers or into other feedstock chemicals, enabling the production of virgin-quality recycled materials.^6^ However, both of these approaches encounter challenges when applied to the chemical recycling of PE. Recycling of PE to its monomer (ethylene) is not feasible due to the absence of functional groups that can serve as reactive sites for depolymerization reactions. As a result, selective cleavage of the polymer chain is nearly impossible under practical conditions.^7^ Recycling of PE into feedstock chemicals, primarily through pyrolysis, is a more common pathway, but it remains a challenging process. The high activation energy (284 kJ/mol) for scission along the PE chain^8^ necessitates very high temperatures, making pyrolysis energy-intensive and less sustainable.

Solvent-based recycling presents a promising alternative to address the limitations of mechanical and chemical recycling. The physicochemical foundation of solvent-based recycling is comparatively straightforward: the target polymer is selectively dissolved and extracted in a chosen solvent, allowing undissolved impurities to be separated by filtration.^9^ The polymer is then recovered by antisolvent addition, solvent evaporation, or cooling.^10^ The advantages of dissolution/precipitation are many-fold:^11^ by optimizing the solvent and polymer recovery conditions, it is possible to achieve selective separation of the target polymer, isolating it from other polymers in a composite or multilayer material, as well as from small molecule additives or contaminants; additionally, degradation can be minimized if high temperatures are avoided during the dissolution process.

In the design of solvent-based recycling processes, solvent selection plays a pivotal role. Effective solvent selection requires evaluating the solvent-specific polymer solubility and dissolution rate, as well as the solvents’ environmental, health, and safety (EHS) profile. While numerous studies on the solvent-based recycling of PE have been published in academic papers^12–17^ and patents^18,19^ they are primarily focused on the use of toluene and xylene as solvents, though elevated temperatures are required (up to 140^◦^ C). Although these solvents are able to dissolve PE, they are often considered hazardous.^20^ Recently, Aparício et al.^21^ reported a novel selective dissolution–precipitation recycling process for high-density polyethylene (HDPE) and polyethylene terephthalate (PET), where HDPE was successfully dissolved in three bioderived solvents: limonene, α-pinene, and *p*-cymene. *p*-Cymene is commonly found in essential oils, particularly those of cumin and thyme.^22^ Another solvent found to effectively dissolve PE is dibutoxymethane (1-(butoxymethoxy)­butane),^23^ a common biodiesel additive due to its high cetane number and low boiling point.^24,25^ It is synthesized from *n*-butanol and formaldehyde^26,27^ both of which can be obtained from renewable sources. *n*-Butanol can be produced via lignocellulosic biomass fermentation^28^ or by reducing volatile fatty acids with hydrogen,^29^ while formaldehyde is typically synthesized from biomethanol. This shift toward bioderived solvents opens a promising pathway for safer and more sustainable PE recycling practices.

There is a pressing need for accurate experimental PE solubility data and reliable predictive models that can be used to broaden the range of compounds that can be considered during solvent selection. However, despite the growing interest in solvent-based recycling, solubility data for PE remain scarce. Existing studies focus on qualitative observations or solubility acquired under limited conditions across a narrow range of solvents, providing insufficient insight into the detailed phase behavior of PE.^30–32^

At fixed pressure, solid–liquid equilibria (SLE) measurements are typically carried out either isothermally by varying the composition (isothermal measurements), or at fixed composition where the temperature is varied (polythermal measurements).^30^ In isothermal measurements, the solubility of a solute is determined by varying the concentration through either “excess solute” or “excess solvent” until full dissolution is achieved.^33^ In this case, the saturated concentration or composition is the examined observable; full dissolution can be assessed either visually by noting when no further dissolution occurs^34^ or by determining the concentration of the saturated solution through optical or spectroscopic analysis of the homogeneous phase. By contrast, polythermal measurements involve maintaining a fixed composition of the solute–solvent mixture while gradually changing the temperature to identify the point of complete dissolution. Temperature is the examined observable, and the onset of phase separation–the reverse of dissolution–is indicated by the cloud point,^35^ the temperature at which the solution becomes turbid. It can be determined either visually or by monitoring changes in turbidity (or its opposite, transmissivity), with both methods relying on the same principle but differing in sensitivity.^31,35,36^

In our current study, an automated multireactor crystallizer, Crystal 16 (Technobis Crystallization Systems, The Netherlands), is employed to perform solubility measurements of PE in several solvents based on the polythermal method. This instrument has seen a number of applications in crystallization^37,38^ and solubility experiments.^39–41^

Amrihesari et al.^42^ demonstrated an example of the application of Crystal 16 in the solubility measurement of polypropylene (PP) and polyethylene glycol (PEG) in toluene, employing both cloud-point detection and the analysis of plateau turbidity to quantify the solubility of the polymers.

We use the clear point instead of the cloud point as the primary indicator of solubility. In contrast to the cloud point, which marks the onset temperature of phase separation, the clear point refers to the temperature during dissolution at which the transmissivity returns to its initial value, indicating complete dissolution. This choice is supported by previous findings,^43^ which demonstrated the presence of hysteresis in cloud point measurements for polymers due to kinetic limitations in phase separation. Although the reported observation of hysteresis relates to a mixture exhibiting lower critical solution temperature (LCST) behavior, it highlights that cloud-point measurements can be condition-sensitive due to kinetic effects. By comparison, the clear point is a more reproducible and thermodynamically meaningful measure of solubility, as it directly relates to the complete dissolution of PE.

While critically needed for the development of novel dissolution processes, the accurate determination of polymer solubility can be expensive and time-consuming. Predictive thermodynamic models play an important role to minimize the number of measurements and enable the exploration of new solvents, including those not yet experimentally characterized. In this context, the choice of solvents based on Hansen solubility parameters (HSPs) has been suggested,^44^ though such empirical parameters cannot be used to predict quantitatively the solubility. More recently, data-driven machine learning (ML) classification algorithms have also emerged as powerful tools for the rapid screening of viable polymer–solvent pairs.^45–47^ For example, recent ML models trained on high-throughput turbidity data have proven highly effective at categorizing polymer solubility behavior.^47^ However, although ML classification models are highly effective for solvent screening, they rely on nonequilibrium data and remain generally unsuitable for predicting rigorous phase equilibria and thermodynamic properties needed in process simulations—such as solid–liquid equilibrium temperatures and liquid and vapor enthalpies under varying process conditions. In recent work, COSMO-RS,^48^ in which a detailed molecular description of polymer–solvent interactions is considered, has been employed to predict the solubility of polymers in solvents for dissolution applications.^34,49^ Using COSMO-RS to predict polymer solubility is computationally demanding, as it requires quantum mechanical calculations to determine representative conformations of the polymer along with an experimentally measured reference solubility data point. Additionally, the validity of this method for the prediction of solubilities across different polymer molecular weights and temperature ranges has been tested only for a small number of solvents.^49^ Lattice cluster theory (LCT) has also been applied as a predictive tool for the SLE of polymer + solvent systems.^50,51^ LCT has been employed to predict the SLE of ethylene/1-octene copolymers in 1,2,4-trichlorobenzene^52,53^ and dibutoxymethane^54^ with good agreement with experimental data. Furthermore, the studies assessed the effects of crystallinity, branching, and molecular weight distribution on the phase behavior of the mixtures.

Molecular equations of state (EoSs) represent an alternative route for predicting the thermodynamic properties of complex mixtures, including polymer systems. The statistical associating fluid theory (SAFT), first developed in the late 1980s,^55,56^ has been widely used to model and predict the thermodynamic behavior of complex fluids.^57,58^ It has a rigorous statistical mechanical foundation based on Wertheim’s perturbation theory^59–62^ and considers the effects of molecular size, shape, and distinct intermolecular interactions, such as hydrogen bonding. The use of the equation of state to model the amorphous polymer as liquid-like has been shown to provide a complete description of the phase behavior of polymer + solvent mixtures, by considering vapor–liquid equilibrium (VLE), liquid–liquid equilibrium (LLE)^63–65^ as well as SLE^66^ when crystalline polymer is present. Ghosh and Chapman^67^ employed the original SAFT model to predict the SLE of 13.6 g/mol PE in degraded PE, paraffin wax, cetene, and *n*-heptane with good agreement with experimental data. The perturbed-chain statistical associating fluid theory (PC-SAFT) EoS,^68^ in which the original SAFT equation is modified by applying the perturbation theory of Barker and Henderson^69^ to a hard-chain reference fluid, has also been used to study polymer + solvent mixtures. Arndt et al.^30^ applied PC-SAFT to predict the solubility of 133 kg/mol PE in *n*-hexane and cyclohexane, finding close agreement to experimental data. Xiong et al.^65^ compared the predictive performance of the original SAFT version with the Sanchez-Lacombe theory in predicting the LCST LLE curves of various PE samples in high-pressure *n*-butane and *n*-pentane. They found that SAFT yielded lower deviations from experimental data. Paricaud et al.^64^ applied the SAFT-VR SW^70^ (square well) version of the equation of state to predict the sorption of *n*-pentane in amorphous PE, via VLE and LLE calculations. They also examined the influence of polymer molecular weight on the cloud curves, reporting good overall agreement with experimental observations. In terms of SLE of polymer + solvent mixtures, Pan and Radosz^71^ studied the phase behavior of semicrystalline polymers with the copolymer SAFT approach.^72^ In this method, segment interactions are described through a Lennard-Jones potential, similar to the original SAFT equation. However, unlike the original SAFT, the copolymer approach incorporates heteronuclear segments to account for copolymers and introduces bond fractions to capture polymer branching density. They employed sensitivity analysis to verify the effects of crystallinity, fusion temperature, molecular weight, and pressure on the phase behavior (SLE and LLE) of mixtures of PE and propane, *m*-xylene and amyl acetate.

In the SAFT variants discussed so far, except for the copolymer SAFT model, which extends the theory to copolymers and branched polymers, the molecular segments are treated as homonuclear. This assumption limits the model’s transferability to systems involving chemically diverse solvent molecules, particularly when experimental data are scarce and parametrization becomes time-consuming. A heteronuclear formulation that incorporates a group-contribution (GC) framework is more suitable for capturing the behavior of a broader range of solvents with fewer parameters.

The SAFT-γ Mie EoS^73–75^ is such an approach, with molecules described as comprising distinct functional groups based on a fused heteronuclear model, with interactions between segments characterized through Mie potentials.^73^ Employing a third-order perturbation expansion for the monomeric term using the Mie potential in the SAFT-γ Mie equation has been shown to deliver accurate thermodynamic properties of complex fluids, including derivative properties.^75–77^ The key strength of the GC method is its ability to predict properties based on known functional group contributions, so that the behavior of any pure component or mixture containing the known functional groups can be predicted. The GC method allows for the description of compounds for which experimental data were not included in parameter development, increasing the transferability of the EoS.

SAFT-γ Mie has already been demonstrated to be a valid model for describing the thermodynamic behavior of polymers.^63,73^ Valsecchi et al.^63^ modeled the thermodynamic properties of water + polyethylene glycol (PEG) mixtures with the SAFT-γ Mie approach. In their work, the calculated orthobaric LLE curves accurately described the experimental cloud points for various high molecular weight PEG samples, showing that the equation of state can describe complex systems such as polymers using molecular interaction parameters obtained from smaller-molecule data. The absorption of hydrocarbons in semicrystalline PE and polypropylene was also modeled by Valsecchi et al.^78,79^ who analyzed the effect of structural properties such as crystallinity on the fluid-phase equilibria of the mixtures. In recent work, SAFT-γ Mie has also been applied to model the solubility of CO_2_ in rubbery and glassy amorphous polystyrene and poly­(methyl methacrylate) (PMMA).^80^

Here, we examine the applicability of the SAFT-γ Mie approach to different PE samples in various solvents that are relevant to solvent-based plastic recycling applications. We compare the calculations to measurements of the temperature-dependent solubility of PE in various solvents obtained using the polythermal method. Four PE samples with varying molecular weight, density, and fusion properties are investigated in solvents selected based on several properties that are relevant to plastic recycling. The results of the current study will deepen the understanding of the dissolution of PE, offering valuable insights for future solvent selection and process design in the solvent-based recycling of PE.

In the next section, we outline the experimental methodology and the SAFT-γ Mie group-contribution model. In the results section, the experimentally measured fusion properties of the four PE samples studied are presented, as well as their measured solubilities in a wide range of solvents. The validation of our SAFT-γ Mie SLE model, PE solubility predictions, and the accuracy of our theoretical calculations are also detailed. In the last section, we present our conclusions.

## Methods

### Materials

Four PE samples were purchased as commercial products, labeled LDPE-1, LDPE-2, LDPE-3, and MDPE. LDPE-1, LDPE-2, and MDPE were purchased from Merck kGaA, Germany. LDPE-3 was purchased from Thermo Fisher Scientific, Germany. All solvents were purchased from Merck kGaA, Germany, at different purities: toluene (≥99.5%), *p*-xylene (≥99.0%), mesitylene (≥98%), *n*-dodecane (≥99%), *p*-cymene (≥99%), limonene (1:1 mixture of D- and L-forms, ≥ 95.0%), decahydronaphthalene or decalin (mixture of cis- and transisomers, ≥ 98%), cyclohexanone (≥99.8%), dibutoxymethane (≥97.0%), and (±)-α-pinene (≥98%). All of the chemicals were used as received.

### Fusion Property Measurements

Fusion properties of the PE samples were determined using differential scanning calorimetry (DSC). The instrument used was a NETZSCH 200 F3 thermal analyzer. The calibration was done with six standard samples (adamantane, bismuth, cesium chloride, indium, tin, and zinc) at a heating/cooling rate of 5 K/min. For each measurement, a weighed amount of polymer powder or pellets was sealed in an aluminum crucible, which was then placed alongside a reference in the measurement chamber. Nitrogen gas was purged through the chamber throughout the experiment to maintain an inert atmosphere.

The temperature program consisted of the following steps: initially, the temperature was raised from 298 to 423 K to fully melt the PE sample, removing any thermal history resulting from prior manufacturing processes. The temperature was then decreased to 273 K to ensure complete solidification. Finally, the sample was reheated to 423 K to measure the temperature of fusion *T* ^fus^ and the specific enthalpy of fusion Δ*h* ^fus^. All heating and cooling rates in this thermal cycle were fixed at 5 K/min, consistent with the calibration conditions.

The temperature of fusion *T* ^fus^ was taken as the onset temperature of the endothermal peak, while Δ*h* ^fus^ was calculated as the area under the endothermal peak, divided by the mass of the PE sample.

### Polythermal Solubility Measurements

The polythermal solubility measurements were conducted using an automated multireactor crystallizer, Crystal 16, from Technobis Crystallization Systems. It is equipped with 16 separate reactors, divided into 4 blocks. In each block, an individual temperature profile can be set. Temperature ramping can be realized in increments of 0.1 K/min with a detection accuracy of 0.5 K. Each reactor is equipped with magnetic stirring and an individual turbidity sensor with a red LED light source, which has a peak output at 645 nm.

The experimental procedure for polythermal solubility measurements was conducted as follows: a precisely weighed amount of PE sample and solvent was loaded into a 1.5 mL glass HPLC vial equipped with either a magnetic stir bar or an overhead stirrer, depending on the anticipated viscosity of the solution. The vial was sealed and placed in the reactor, which was then rapidly heated (heating rate of 20 K/min) to 5 K below the temperature of fusion of the respective PE sample (375.2–399.5 K) to facilitate rapid dissolution while ensuring that a solid polymer phase may, in principle, be stable. This temperature was held until complete dissolution, which was confirmed by visual inspection. This initial dissolution step was intended to eliminate kinetic limitations arising from polymer morphology and thermal history, ensuring reproducible acquisition of the thermodynamic clear point in subsequent cycles. At this stage, the transmissivity was calibrated to 100%, thus setting the fully dissolved solution as a reference. This calibration accounted for possible optical variations such as diffraction, absorption, and refraction caused by the polymer, solvent, and the measurement vial. By setting this baseline of 100%, the clear point in the following thermal cycles could be consistently identified as the temperature at which the transmissivity returned to this calibrated value, indicating complete dissolution.

Before starting the measurement cycles, a prerun cycle was conducted to set the temperature range of the cycles as follows: the temperature was decreased at a cooling rate of 3 K/min until the transmissivity dropped from 100%, i.e., the cloud point was reached. Phase separation was confirmed visually. The temperature was then increased at a heating rate of 3 K/min until the transmissivity returned to 100%, i.e., the clear point was reached. Visual confirmation of the complete dissolution was conducted again.

Subsequently, measurement cycles were undertaken: the temperature was decreased to 2 K below the cloud point (minimum temperature of the cycle) and then raised to 2 K above the clear point (maximum temperature of the cycle), as approximately determined by the prerun cycle. This thermal cycle was repeated at least three times. The average of the measured clear points of the first three cycles was taken as the representative value of the SLE temperature.

### Hansen Solubility Parameter (HSP) Calculations

In the Hansen solubility parameter (HSP) framework, a substance is characterized by three parameters, representing the energy associated with dispersion interactions δ_*d*_, dipole interactions δ_*p*_, and hydrogen bonding interactions δ_*h*_.^81^ It is assumed that the solubility of a solute in a solvent is dependent on the similarity of these three interaction parameters between the two substances, which is quantified by the geometric distance *R*_*a*_ between the HSP values of the solute (1) and the solvent (2);^81^ i.e.,

1 $$R_{a}=\sqrt{4{\left(\delta_{d1}-\delta_{d2}\right)}^{2}+{\left(\delta_{p1}-\delta_{p2}\right)}^{2}+{\left(\delta_{h1}-\delta_{h2}\right)}^{2}}$$

In addition, a characteristic value, known as the interaction radius *R*_0_, is specified for each solute. The 4 parameters: δ_*d*_, δ_*p*_, δ_*h*_, and *R*_0_ can be found in the HSP database.^82^ If the distance *R*_*a*_ between the solute and the solvent in the Hansen space is smaller than the solute’s interaction radius *R*_0_, or in other words, if

2 $$R_{a}/R_{0}<1$$

then the solute is assumed to be dissolved in the solvent and vice versa. The ratio *R*_*a*_/*R*_0_, also referred to as the Relative Energy Difference (RED), provides a useful measure for *a priori* solvent selection. In the current work, the RED value is used to select an initial set of solvents (cf. Supporting Information (SI) Table S1).

### SAFT-γ Mie Model and Theory

In the SAFT-γ Mie approach, the Helmholtz free energy, *A*, of a fluid mixture of nonionic species, is written as the sum of four distinct contributions^73,74,83,84^

3 $$A=A^{\mathrm{ideal}}+A^{\mathrm{mono}}+A^{\mathrm{chain}}+A^{\mathrm{assoc}}$$

where *A* ^ideal^ is the ideal gas free energy, *A* ^mono^ represents the contribution related to the Mie segment–segment interactions (repulsion and dispersion), *A* ^chain^ is the free energy that accounts for the formation of molecules from Mie segments, and *A* ^assoc^ denotes the contribution to the free energy from association interactions (hydrogen bonding).

In the SAFT-γ Mie EoS, molecules are modeled as heteronuclear chains of fused spherical segments with attractive associative sites.^75^ Each compound, *i*, is characterized by the number of groups it contains, *v*_*k,i*_, where *k* ∈ *G*, the set of all functional groups. A functional group *k* consists of $v_{k}^{*}$ identical fused spherical segments. In Figure 1, examples of SAFT-γ Mie models of the compounds examined in this study are presented, with each functional group represented by a distinctly colored sphere. Interactions between segments in groups *k* and *l* are described by the Mie^85^ potential, given as

4 $$\Phi_{kl}^{\mathrm{Mie}}\left(r_{kl}\right)=C_{kl}\varepsilon_{kl}\left[{\left(\frac{\sigma_{kl}}{r_{kl}}\right)}^{\lambda_{kl}^{r}}-{\left(\frac{\sigma_{kl}}{r_{kl}}\right)}^{\lambda_{kl}^{a}}\right]$$

where *r*_*kl*_ is the center-to-center distance between *k* and *l*, σ_*kl*_ is a size parameter corresponding to the segment’s diameter in instances of like interactions (*k* = *l*), *ε*_*kl*_, the dispersion energy, indicates the depth of the potential well, and $\lambda_{kl}^{r}$ and $\lambda_{kl}^{a}$ describe the repulsive and attractive ranges of the potential, respectively. The prefactor *C*_*kl*_ is given by

5 $$C_{kl}=\frac{\lambda_{kl}^{r}}{\lambda_{kl}^{r}-\lambda_{kl}^{a}}{\left(\frac{\lambda_{kl}^{r}}{\lambda_{kl}^{a}}\right)}^{\lambda_{kl}^{a}/(\lambda_{kl}^{r}-\lambda_{kl}^{a})}$$

to ensure the minimum value of $\Phi_{kl}^{\mathrm{Mie}}\left(r_{kl}\right)$ is −*ε*_*kl*_. In order to determine the proportion of total free energy and other thermodynamic properties contributed by a specific segment *k* in a molecule, a shape factor *S*_*k*_ is included as an additional parameter.^75^

**Figure 1:**
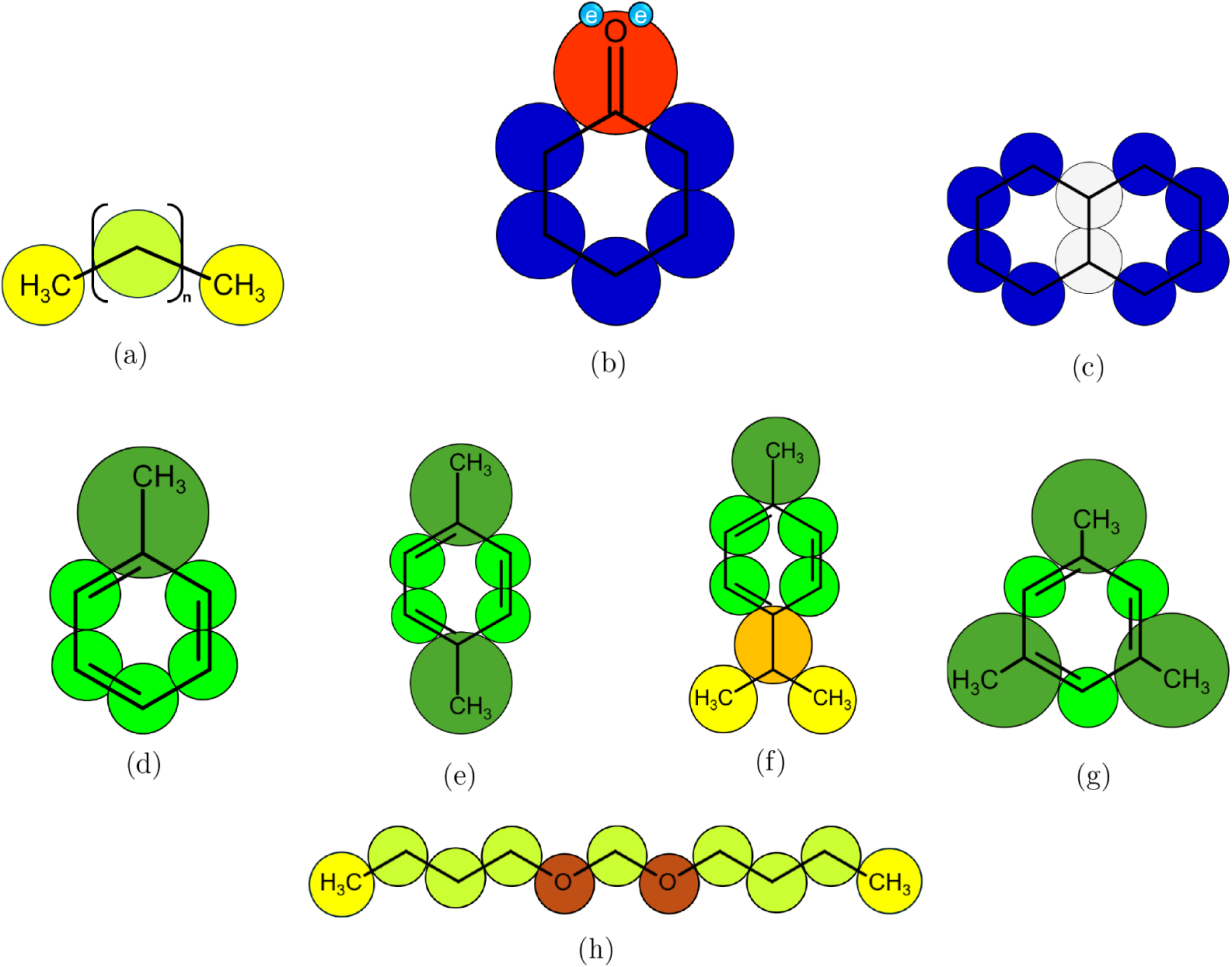
Molecular representations and SAFT-γ Mie molecular models of the compounds considered in the current work: (a) *n*-alkanes, PE, (b) cyclohexanone, (c) decalin, (d) toluene, (e) *p*-xylene, (f) *p*-cymene, (g) mesitylene, and (h) dibutoxymethane. PE is characterized as an *n*-alkane with *n* repeating CH_2_ groups. (Segments are not presented to scale).

Combining rules can sometimes be used to characterize cross-interaction parameters between two unlike (*k*≠*l*) groups. These offer an initial estimate, although in practice these parameters are fine-tuned using experimental data. An exception to this is the unlike segment diameter σ_*kl*_, which is always evaluated using the Lorentz combining rule:^86^

6 $$\sigma_{kl}=\frac{\sigma_{kk}+\sigma_{ll}}{2}$$

The unlike attractive and repulsive exponent $\lambda_{kl}^{a}$ and $\lambda_{kl}^{r}$ , respectively, are calculated as^73^

7 $$\lambda_{kl}^{m}=3+\sqrt{\left(\lambda_{kk}^{m}-3\right)\left(\lambda_{ll}^{m}-3\right)},,m\in \left\{a,r\right\}$$

The unlike dispersion energy *ε*_*kl*_ is computed by applying the Berthelot-like geometric-mean rule, which also considers size asymmetry^73^

8 $$\varepsilon_{kl}=\frac{\sqrt{\sigma_{kk}^{3}\sigma_{ll}^{3}}}{\sigma_{kl}^{3}}\sqrt{\varepsilon_{kk}\varepsilon_{ll}}$$

For highly polar interactions, e.g., hydrogen bonding, further off-center square-well association sites are embedded in specific segments as required. The square-well interaction between an association site of type *a* on segment *k* and a site of type *b* on segment *l* is defined as,

9 $$\Phi_{kl,ab}^{\mathrm{HB}}\left(r_{kl,ab}\right)=\left\{ \begin{array}{ll} -\varepsilon_{kl,ab}^{\mathrm{HB}} & \mathrm{if}r_{kl,ab}\leq r_{kl,ab}^{c}\\ 0 & \mathrm{if}r_{kl,ab}>r_{kl,ab}^{c} \end{array}\right.$$

where *r*_*kl,ab*_ is the distance between the centers of the two sites, $-\varepsilon_{kl,ab}^{HB}$ is the association energy, and $r_{kl,ab}^{c}$ is the cutoff range of the interaction. Site *a* is located at a distance $r_{kk,aa}^{d}$ from the center of segment *k* (parameters are similarly defined for site *b* on segment *l*). In the current version of the theory, $r_{kk,aa}^{d}$ and $r_{kl,ab}^{c}$ are incorporated via a bonding volume parameter $K_{kl,ab}^{\mathrm{HB}}$ .^73^ Following Wertheim’s thermodynamic perturbation theory (TPT1),^87^ the contributions from bonding at individual sites are considered independent. This means that the relative positions of the various sites on a segment are not considered. To characterize an associating group *k* fully, the number $N_{\mathrm{ST},k}$ of distinct site types and the number of sites of each type, e.g., $n_{k,a},n_{k,b},...,n_{k,N_{\mathrm{ST},k}}$ are specified.

The unlike association parameters $\varepsilon_{kl,ab}^{\mathrm{HB}}$ and $K_{kl,ab}^{\mathrm{HB}}$ can be calculated by combining rules as follows:^74^

10 $$\varepsilon_{kl,ab}^{\mathrm{HB}}=\sqrt{\varepsilon_{kk,ab}^{\mathrm{HB}}\varepsilon_{ll,ab}^{\mathrm{HB}}}$$

and

11 $$K_{kl,ab}^{\mathrm{HB}}={\left(\frac{\sqrt[{3}]{K_{kk,ab}^{\mathrm{HB}}}+\sqrt[{3}]{K_{ll,ab}^{\mathrm{HB}}}}{2}\right)}^{3}$$

Values for the like and unlike group interaction parameters used in the current work are provided in Haslam et al.^75^ and Paliwal et al.^88^ The Helmholtz free energy, which is a function of temperature *T*, volume *V* and the vector of moles **N**, can be used to obtain other thermodynamic properties through standard thermodynamic relations. In this study, the relations to calculate pressure *P*, chemical potential μ_*i*_ and fugacity coefficient *φ̂*_*i*_ are of particular relevance. The pressure *P* is obtained as

12 $$P=-{\frac{\partial A\left(T,V,N\right)}{\partial V}|}_{T,N}$$

the residual chemical potential of *i* as

13 $$\mu_{i}^{\mathrm{Res}}\left(T,P,N\right)={\frac{\partial A^{\mathrm{Res}}(T,V,N)}{\partial N_{i}}|}_{T,V,N_{j\neq i}}-RT\ln Z\left(T,P,N\right)$$

and the fugacity coefficient as

14 $$\ln{\mathrm{\varphi ̂}}_{i}\left(T,P,N\right)=\frac{\mu_{i}^{\mathrm{Res}}\left(T,P,N\right)}{RT}$$

Here, *A* ^Res^ represents the difference between the free energy *A* and the ideal free energy *A* ^ideal^, *Z* is the compressibility factor calculated as $Z=Pv_{P}/RT$ , where *v*_*P*_ is the molar volume at the given pressure defined as $v_{P}=V/N$ , *N* is the total number of moles. These properties are used to calculate fluid-phase equilibria.

In the SAFT-γ Mie GC approach, PE comprises two CH_3_ groups and a number $n_{{\mathrm{CH}}_{2}}$ of identical CH_2_ groups derived from the molecular weight of the given PE.^73^ While 10 solvents were studied experimentally, those 8 for which all required SAFT-γ Mie groups have been previously defined in the parameter matrix^75^ are considered. The SAFT-γ Mie group characterization for these 8 modeled solvents is given in Table 2. Figure 2 details the specific group interaction submatrix relevant to this study. It incorporates established parameters from prior research,^75^ alongside interactions derived from combining rules (CR) that fall outside the focus of our current analysis.

**Figure 2:**
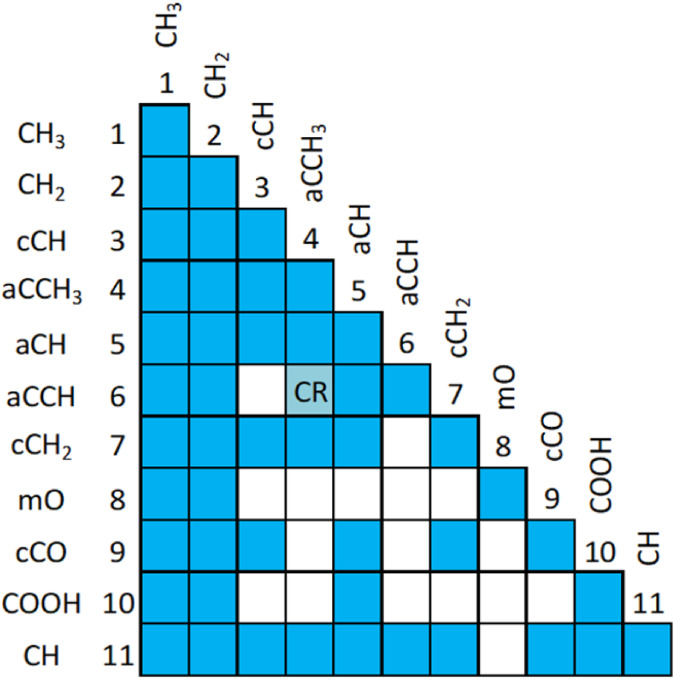
Summary of the group interactions employed to model PE solubility in eight solvents with the SAFT-γ Mie EoS. Blue shading indicates that the group interaction parameters have been optimized and published in previous work. Light blue shading denotes unlike interactions for which parameters have not been refined, and which are calculated using CR.^73^ White squares highlight unoptimized parameters that are not needed in the current work. Parameter tables are available in the SI (cf. Table S6).

### Solid–Liquid Equilibria

At a given *T* and *P*, the solubility of a pure solid solute *i* in a solvent is defined as the maximum amount of the solute that can be dissolved in a given amount of solvent to form a homogeneous solution. The solubility equation, neglecting the entropic terms,^75^ is given as

15 $$\ln x_{i}^{L}\left(T,P\right)=-\frac{\Delta h_{i,m}^{\mathrm{fus}}\left(T_{i}^{\mathrm{fus}},P\right)}{R}\left(\frac{1}{T}-\frac{1}{T_{i}^{\mathrm{fus}}}\right)-\ln\gamma_{i}^{L}\left(T,P,x^{L}\right)$$

where $x_{i}^{L}$ is the mole fraction of solute *i* in the liquid phase, $\Delta h_{i,m}^{\mathrm{fus}}$ is the specific molar enthalpy of fusion of *i* $\left(\Delta h_{i,m}^{\mathrm{fus}}=M_{w,i}\Delta h_{i}^{\mathrm{fus}}\right)$ , *M*_*w,i*_ is the molecular weight of solute *i*, $\Delta h_{i}^{\mathrm{fus}}$ is the specific mass enthalpy, $T_{i}^{\mathrm{fus}}$ is the temperature of fusion of *i*, *R* is the universal gas constant, and $\gamma_{i}^{L}$ the activity coefficient of *i* in the liquid phase calculated as

16 $$\gamma_{i}^{L}\left(T,P,x^{L}\right)=\frac{{\mathrm{\varphi ̂}}_{i}\left(T,P,x^{L}\right)}{{\mathrm{\varphi ̂}}_{i}^{*}\left(T,P\right)}$$

where *φ̂*_*i*_(*T*, *P*, **x** ^**L**^) and ${\mathrm{\varphi ̂}}_{i}^{*}\left(T,P\right)$ are the fugacity coefficients of *i* in the liquid mixture and of the pure *i* in the liquid phase at the same *T* and *P* and $x^{L}=\frac{N^{L}}{N^{L}}$ is the liquid phase mole fraction vector.

As in previous treatments of semicrystalline polymers with SAFT-γ Mie,^79^ we define the crystallinity (*c*) of the polymer as the ratio between the specific enthalpies of fusion of the semi and fully crystalline polymers, used when the experimental Δ*h* ^fus^ is unavailable:

17 $$c=\frac{\Delta h_{i}^{\mathrm{fus}}\left(T_{i}^{\mathrm{fus}},P\right)}{\Delta h_{i,c}^{\mathrm{fus}}\left(T_{i,c}^{\mathrm{fus}},P\right)}$$

where $\Delta h_{i}^{\mathrm{fus}}$ is the specific enthalpy of fusion of the semicrystalline polymer. The specific enthalpy of fusion of the fully crystalline PE, $\Delta h_{i,c}^{\mathrm{fus}}\left(T_{i,c}^{\mathrm{fus}},P\right)=293.02J/g$ , is the measured enthalpy of fusion of fully crystalline PE reported in the literature^89^ and is assumed to be independent of molecular weight. In our model, we describe PE as containing 2 × CH_3_ groups and $n_{{\mathrm{CH}}_{2}}\times {\mathrm{CH}}_{2}$ groups. The number of repeating CH_2_ groups is calculated from the molecular weight of the polymer sample as

18 $$n_{{\mathrm{CH}}_{2}}=\frac{M_{w,\mathrm{PE}}-2M_{w,{\mathrm{CH}}_{3}}}{M_{w,{\mathrm{CH}}_{2}}}$$

### Quantifying the Accuracy of the Model

In order to quantify the agreement between the SAFT-γ Mie predictions and experimental data, we calculate the percentage absolute average deviation (%AAD) of a property *p* for a system (mixture) *s*,

19 $$\%{\mathrm{AAD}}_{s,p}=\frac{1}{N_{s,p}^{\mathrm{tot}}}\overset{N_{s,p}^{\mathrm{tot}}}{\underset{i=1}{\sum }}\left|\frac{X_{s,p,i}^{\exp}-X_{s,p,i}^{\mathrm{calc}}}{X_{s,p,i}^{\exp}}\right|\times 100$$

and the absolute average deviation (AAD),

20 $${\mathrm{AAD}}_{s,p}=\frac{1}{N_{s,p}^{\mathrm{tot}}}\overset{N_{s,p}^{\mathrm{tot}}}{\underset{i=1}{\sum }}\left|X_{s,p,i}^{\exp}-X_{s,p,i}^{\mathrm{calc}}\right|$$

where $N_{s,p}^{\mathrm{tot}}$ is the total number of experimental data points for system *s* and property *p*, $X_{s,p,i}^{\exp}$ is the *i* ^th^ measured value of property *p* of system *s*, and $X_{s,p,i}^{\mathrm{calc}}$ is the respective value calculated with SAFT-γ Mie.

## Results and Discussion

### Characterization of Polymer Samples

The key properties of the four PE samples used in our work are summarized in Table 1. The PE samples vary in density (from low-density to medium-density), molecular weight, polydispersity index, temperature, and enthalpy of fusion. As the molecular weight and melting properties were not provided by the manufacturer for most samples, they were measured as part of the current work. The temperature and enthalpy of fusion were measured by DSC. As can be seen in Table 1, the measured temperatures of fusion *T* ^fus^ lie between 375.2 and 399.5 K, while the measured enthalpies of fusion Δ*h* ^fus^ lie between 89.3 and 201.5 J/g. The molecular weights of the PE samples were measured by high-temperature gel permeation chromatography (HT-GPC). The number-averaged molecular weights *M*_*n*_, of the PE samples range between 1.5 and 22 kg/mol, while the weight-averaged molecular weights ***M*_*w*_**, range between 4.9 and 105 kg/mol. $n_{{\mathrm{CH}}_{2}}$ values for the PE samples were calculated from the measured *M*_*w*_ using eq 18.

**Table 1:** Specifications and Measured Properties of the PE Samples Considered^a^

Notation^b^	Density (g/cm^3^)	*M*_*n*_ (kg/mol)	*M*_*w*_ (kg/mol)	*T* ^fus^ (K)	Δ*h* ^fus^ (J/g)	$n_{{\mathrm{CH}}_{2}}$
LDPE-1	0.92	1.5*	4.9*	375.2*	89.3*	384
LDPE-2	0.925	16*	105*	**389.0**, 379.9*	123.0*	7848
LDPE-3	0.94	22	76	399.5*	201.5*	5416
MDPE	0.94	2.7*	7.6*	382.0–384.0, 378.5*	114.7*	540

a The data were obtained from Merck kGaA, Germany,^90^ except for those indicated with an *, which were measured in the current work. The enthalpy of fusion of fully crystalline PE, Δ*h* ^fus^=293.0 J/g, isused in the modelling. The *T* ^fus^ values employed in the modelling are highlighted in bold.

b The notation (LDPE, MDPE) follows the product names assigned by the providers.

### Solvent Selection

The selection of solvents was performed following the systematic procedure shown in Figure 3 and involved four criteria:

1 HSP criterion. The RED value must be less than one, when calculated using LDPE as the solute, for which the parameter values were taken from Hansen’s original database: δ_*d*_ = 15.9, δ_*p*_ = 4.9, δ_*h*_ = 1.5, and the interaction radius *R*_0_ = 8.0.^82^
2 Boiling-point criterion. The normal boiling temperature of the solvent must be sufficiently high for operational purposes, because the dissolution of PE is usually slow and occurs at elevated temperatures. A value of 338 K (the boiling point of THF) was set as the minimum normal boiling point criterion.
3 Hazard criterion. Solvents that are commonly known to raise toxicity/or environmental concerns should be excluded, as using harmful solvents would contradict the aims of sustainable polymer recycling.
4 Solubility criterion. Complete dissolution of LDPE-1 must be achieved after 24 h of isothermal stirring when mixing LDPE-1 with the solvent at 0.01 g/g weight fraction and heating the mixture to 10 K below its boiling temperature, up to a maximum of 413.2 K. This is performed to avoid excluding solvents with slow dissolution kinetics and to approach SLE.

**Figure 3:**
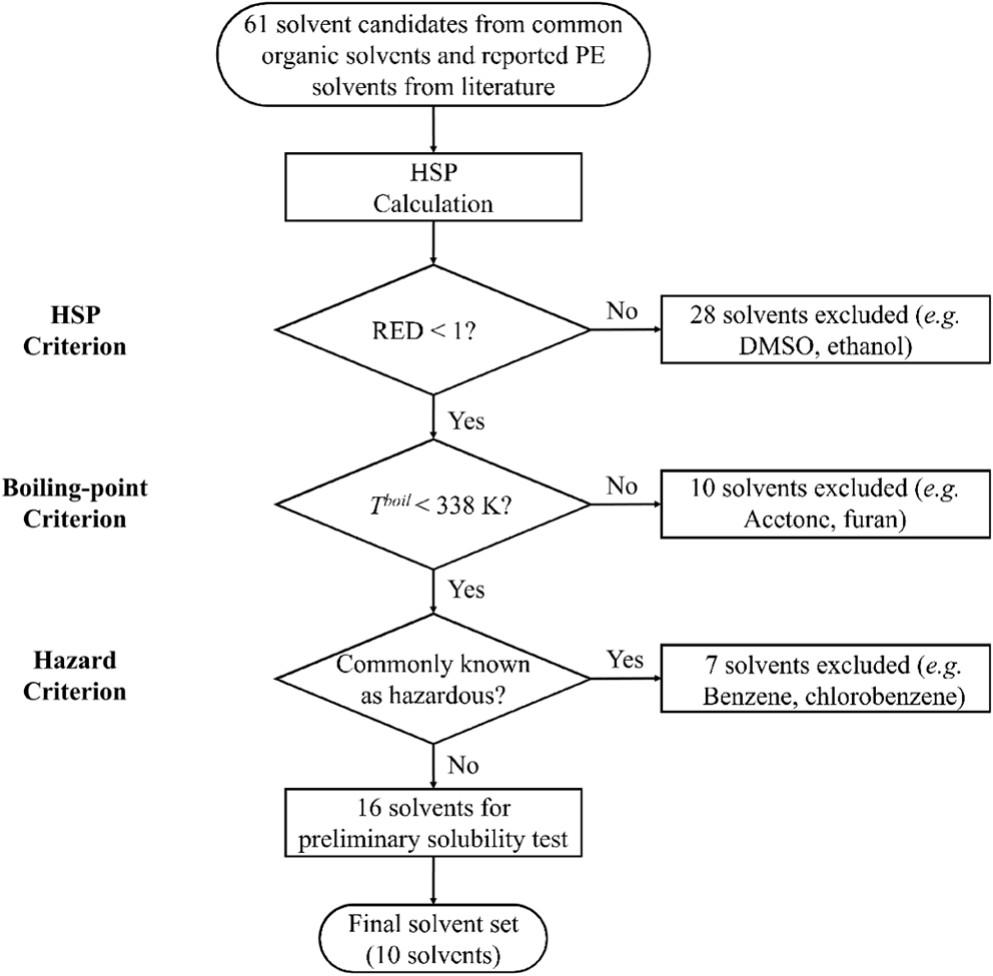
Flowchart of the solvent selection process.

A pool of 61 solvents (cf. SI Table S1) was chosen from commonly used organic solvents, bio-derived solvents, and solvents previously reported in the literature for PE.^12–19,21^ The HSP values were calculated, and it was found that 33 solvents had a RED value lower than one. These 33 solvents were further reduced down to 23 following the boiling-point criterion and 16 following the hazard criterion. For example, low-boiling-point solvents (e.g., acetone, dichloromethane) were excluded due to criterion 1, and solvents that are known to be particularly hazardous (e.g., benzene, chlorinated hydrocarbons such as trichlorobenzene and chloroform) were excluded due to criterion 2.

After the solubility criterion was applied, 10 solvents were selected and used to measure and model the solubility of PE. They are *n*-dodecane, decalin, dibutoxymethane, *p*-cymene, limonene, mesitylene, cyclohexanone, α-pinene, *p*-xylene, and toluene. The structure and properties of the selected solvents, their RED values, and the SAFT-γ Mie groups used to model them, are listed in Table 2. For solvents with stereochemistry, racemic mixtures were used in the experiments, as they are considerably less expensive and are expected to show minimal differences in dissolution performance compared to pure enantiomers.

**Table 2:** Summary of the Properties and RED Values of the Solvents Selected for Solubility Experiments and Their SAFT-*γ* Mie Group Characterization^a^

Name	Molecular formula	CAS Number	SAFT-γ Mie modeling	RED	Normal boiling point (K)^91^
*n*-Dodecane	C_12_H_26_	112–40–3	CH_3_ (× 2), CH_2_ (× 10)	0.78	489.5
Decalin	C_10_H_18_	91–17–8	cCH_2_ (× 8), cCH (× 2)	0.83	469.0(*cis-*)
Dibutoxymethane	C_8_H_18_O_2_	2568–90–3	CH_3_ (× 2), CH_2_ (× 7), mO (× 2)	0.32	452.4
*p*-Cymene	C_10_H_14_	99–87–6	aCH (× 4), aCCH_3_, aCCH, CH_3_ (× 2)	0.48	450.2
Limonene	C_10_H_16_	138–86–3	−	0.62	451.2
Mesitylene	C_9_H_12_	108–67–8	aCH (× 3), aCCH_3_ (× 3)	0.81	437.9
Cyclohexanone	C_6_H_10_O	108–94–1	cCH_2_ (× 5), cCO	0.68	428.6
α-Pinene	C_10_H_16_	80–56–8	−	0.50	429.2
*p*-Xylene	C_8_H_10_	106–42–3	aCH (× 4), aCCH_3_ (× 2)	0.68	411.5
Toluene	C_7_H_8_	108–88–3	aCH (× 5), aCCH_3_	0.69	383.8

a The hyphen (−) denotes that some of the SAFT-*γ* Mie group parameters are unavailable for the compounds.

### Experimental Solid–Liquid Equilibrium of PE + Solvent Mixtures

It is known that the dissolution of polymers can occur at an extremely low rate.^92^ Thus, the effect of the heating rate was investigated first to ensure the consistency and reliability of clear-point acquisition. For this purpose, each of the four PE samples was prepared in decalin and *n*-dodecane at polymer weight fractions of 0.02 and 0.15 g/g, respectively. These two solvents were selected based on preliminary experiments, which identified decalin mixtures as having the lowest SLE temperature at a given weight fraction, indicating the highest PE solubility, while PE + *n*-dodecane exhibited the highest SLE temperature, reflecting the lowest solubility. Experiments were conducted with a fixed cooling rate (0.3 K/min) and a varying heating rate across a minimum of three repeating cycles (from 0.1 to 0.5 K/min). The stirring rate was set to 700 rpm to ensure uniform mixing and rapid heat transfer. It was observed that heating rates below 0.5 K/min produced consistent clear-point results, indicating that equilibrium was reached under this condition. Higher heating rates were observed to cause shifts of the clear point to higher temperatures due to delayed equilibration. Based on this, a heating rate of 0.1–0.2 K/min was chosen throughout the measurements to ensure data reliability.

In Figure 4, the measured solubility-temperature profiles of the four PE samples in the ten solvents are presented. As can be seen in the figure, most data are limited to a weight fraction of around 0.30–0.35 g/g, as higher concentrations resulted in excessive viscosity, preventing effective stirring and reliable measurements. As polymers have a high molecular weight, we use a mass-based concentration

21 $$w_{i}=\frac{x_{i}^{L}M_{w,i}}{\underset{i^{\prime }=1}{\sum }x_{i^{\prime }}^{L}M_{w,i^{\prime }}}$$

where *w*_*i*_ is the weight fraction of component *i* in the mixture.

**Figure 4:**
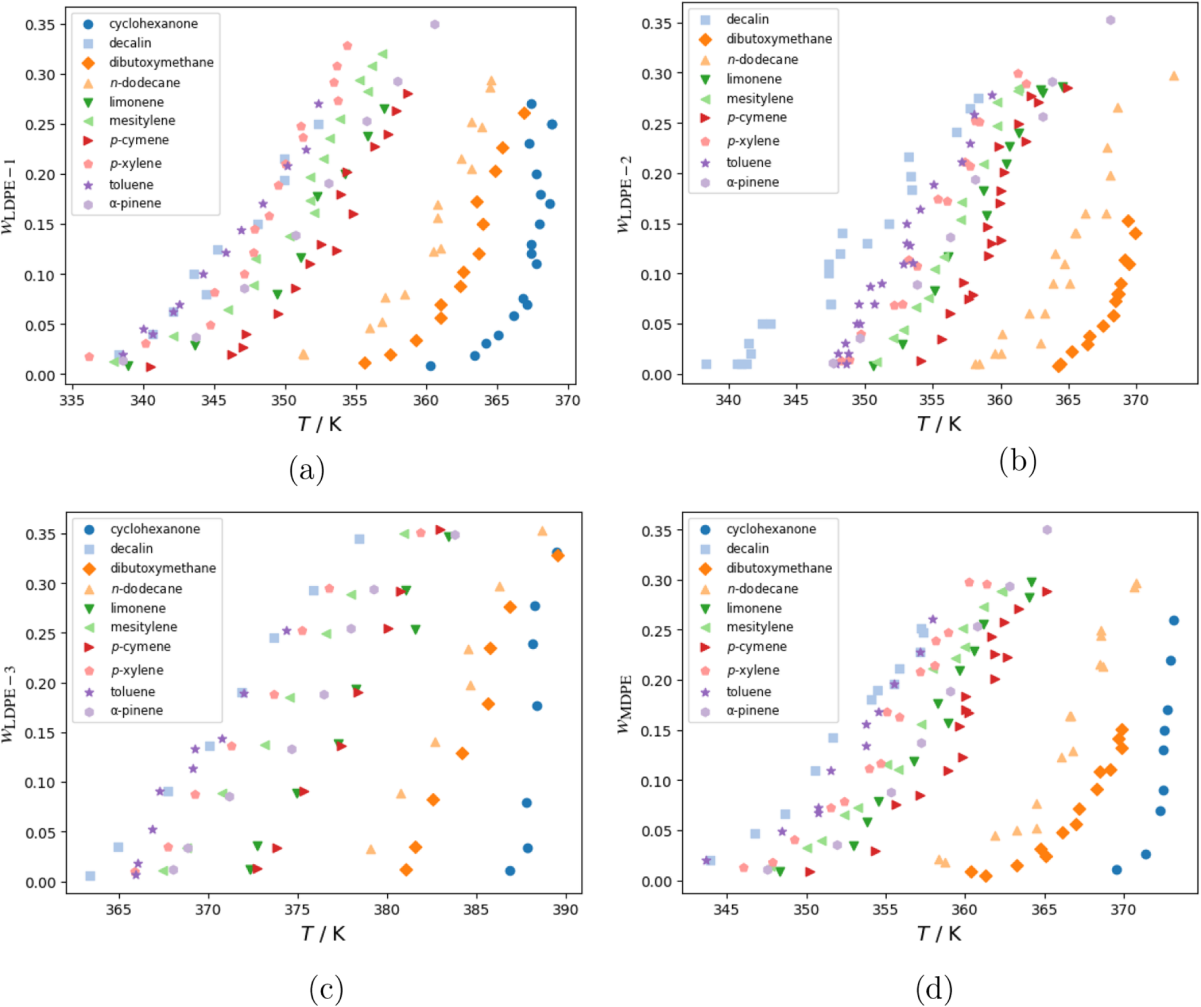
Solubility (in weight fraction)—temperature profiles of the four PE samples in the selected solvents: (a) LDPE-1, (b) LDPE-2, (c) LDPE-3, and (d) MDPE. The dissolution of MDPE and LDPE-2 in dibutoxymethane was not possible beyond a weight fraction of 0.15, regardless of temperature. LDPE-2 did not dissolve in cyclohexanone at 373.5 K after 24 h of stirring. Numerical data can be found in the SI.

A key observation from the experimental results is that, across all the PE-solvent combinations, the SLE temperatures lie within a narrow range (340 to 370 K for LDPE-1, LDPE-2, and MDPE, or 365 to 390 K for LDPE-3), demonstrating the validity of the initial solvent selection using HSPs. The measured PE solubilities in the solvents considered exhibit similar trends with temperature, particularly for a given PE sample. To illustrate better the similarity in the solubility-temperature dependence across PE samples, the solubility—temperature profiles of the four PE samples in toluene and *n*-dodecane are shown in Figure 5.

**Figure 5:**
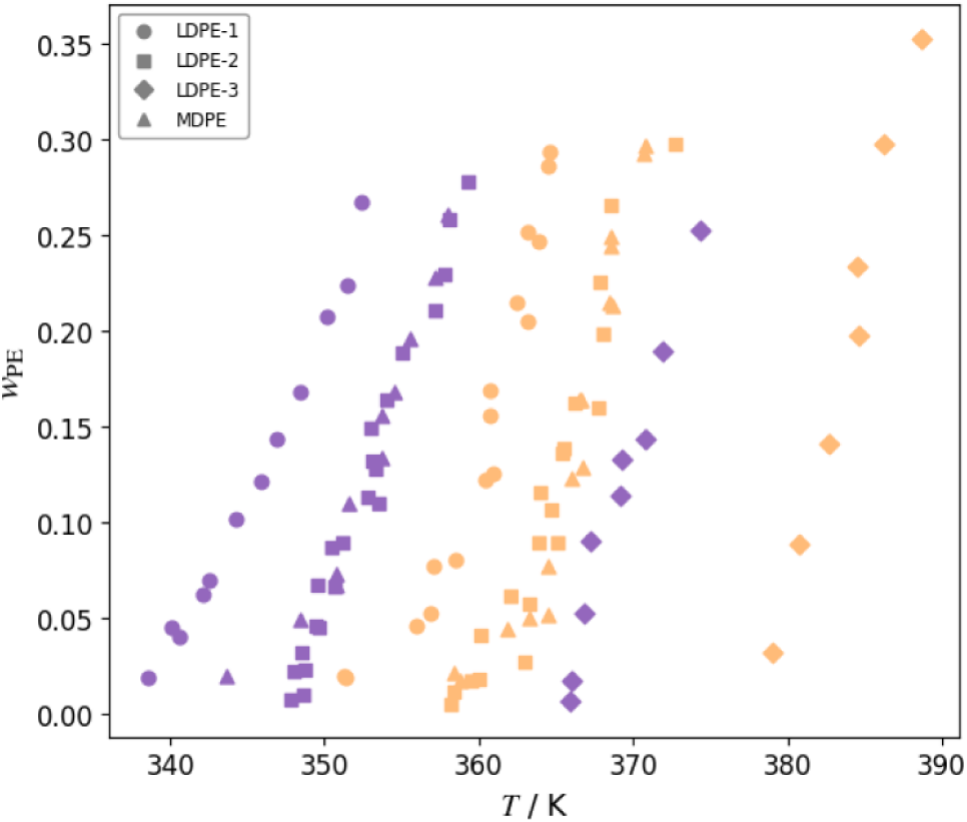
Weight fraction—temperature solubility profiles of the four PE samples in toluene (in purple) and *n*-dodecane (in orange).

Comparing LDPE-1 and LDPE-2, it can be seen in Table 1 that they have similar densities (0.92 vs 0.925 g/cm^3^) but differ significantly in molecular weight (4.9 vs 105 kg/mol) and present small differences in the temperature and specific enthalpy of fusion (375.2 K, 89.3 J/g vs 379.9 K, 123.0 J/g). Comparison of their SLE temperatures in the various solvents (Figures 4 and 5) reveals a 10 K difference at a given weight fraction for all solvents. When comparing the properties of LDPE-2 and MDPE, we find that their melting properties are in a similar range (379.9 K, 123.0 J/g vs 378.5 K, 114.7 J/g), but they differ in molecular weight (105 vs 7.6 kg/mol) and density (0.92 vs 0.94 g/cm^3^). Despite these differences, the measured solubility temperatures of LDPE-2 and MDPE are found to be close in value at a given weight fraction. These observations suggest that the temperature and enthalpy of fusion of PE have a greater influence on the solubility of PE than molecular weight and density. This conclusion is further supported by the significantly higher SLE temperatures observed in the mixtures with LDPE-3 compared to those with other PE samples. Across all solvents, LDPE-3 exhibits SLE temperatures approximately 10–20 K higher than those of other PE samples at a given weight fraction, consistent with the higher temperature of fusion of LDPE-3 (cf. Table 1).

Examination of the measured solubilities reveals that the solvent ranking remains fairly consistent across the four PE samples, as would be expected. In Figure 6, a heat map illustrating the measured SLE temperatures of the four PE samples in the solvents at weight fractions of 0.05, 0.15, and 0.25 g/g, respectively, is presented.

**Figure 6:**
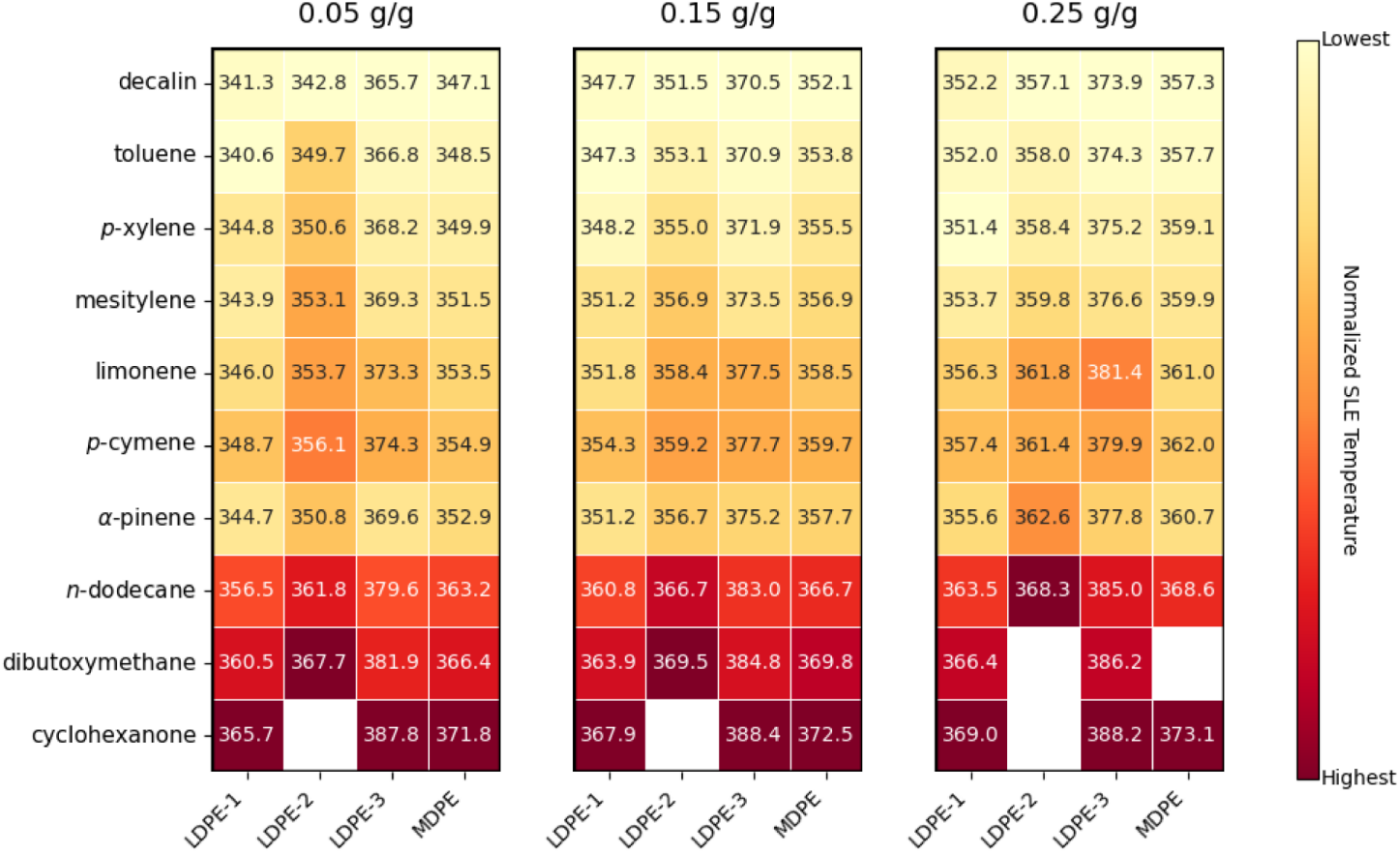
Heat map of the measured SLE temperatures of the four PE samples in the 10 solvents considered, at PE weight fractions of 0.05, 0.15, and 0.25 g/g. Temperature values in the squares are reported in Kelvin (K). Empty squares indicate that no data were acquired for the corresponding PE sample-solvent combination at that weight fraction.

SLE temperatures corresponding to weight fractions of 0.05, 0.15, and 0.25 g/g are determined by interpolating linearly from the two nearest experimental data points. The resulting temperatures for a given PE sample at a given weight fraction are then normalized based on the maximum and minimum values across all solvents, and a graded color scheme in 10 intervals is applied to emphasize visually the differences in SLE temperatures for different solvents.

An analysis of the heat-map uncovers a pattern of solvent performance in the four PE samples. First, the ranking of the solvents remains largely consistent for various PE samples. Decalin, a bicyclic molecule, is found to be the best solvent, with the lowest SLE temperature recorded for any given composition. Aromatic solvents such as toluene and *p*-xylene, two widely reported solvents for PE dissolution,^14,17,93^ along with their structural analogue mesitylene, consistently exhibit an SLE temperature slightly above that of decalin.

Interestingly, the three reported bioderived solvents for PE^21^—limonene, *p*-cymene, and α-pinene—exhibit only slightly higher SLE temperatures than toluene and *p*-xylene, suggesting their potential as alternatives with comparable solubility performance. *n*-Dodecane, despite its structural similarity to PE, performs less effectively in dissolving all of the PE samples. Finally, dibutoxymethane and cyclohexanone, though having low RED values, exhibit the poorest solubility performance. Notably, they fail to dissolve LDPE-2 and MDPE at higher weight fractions.

### SAFT-γ Mie Modeling

We start by evaluating the effects of temperature of fusion and specific enthalpy of fusion, crystallinity and molecular weight of PE, on the SAFT-γ Mie-predicted SLE of PE in selected solvents. We then analyze the predictive capability of SAFT-γ Mie for different solvent types and PE samples. LDPE-1 and LDPE-2 are chosen as representative polymer samples for detailed investigation, as they have the lowest and highest *M*_*w*_ values, respectively (cf. Table 1). The SAFT-γ Mie predictions for MDPE and LDPE-3 are included in the SI (cf. Figures S4–S8). The modeling section concludes with an assessment of the accuracy of the SAFT-γ Mie model by comparing the predictions to the solubility data measured in the current work and to data available in the literature.

### Influence of Properties of Fusion, Crystallinity, and Molecular Weight on PE Solubility Predictions

Eq 15 is used to calculate the solid–liquid solubility of PE in selected solvents. We first study the impacts of the temperature of fusion, $T_{i}^{\mathrm{fus}}$ , specific enthalpy of fusion, $\Delta h_{i}^{\mathrm{fus}}$ , and crystallinity of polymer sample *i*. When considering mixtures for which experimental data were measured in the current work, the pressure *P* is set to the predicted solvent saturated vapor pressure $\left(P_{\mathrm{sol}}^{\mathrm{vap}}\right)$ as our measurements were performed in sealed vials. The pressure, however, is not expected to significantly influence the SLE predictions.^66^

LDPE-2 is chosen as the first sample polymer, and four values of the temperature of fusion are considered: that of the fully crystalline polyethylene, reported as $T_{\mathrm{LDPE},c}^{\mathrm{fus}}=414.6K$ ^94^ for 1.4 × 10^7^ g/mol PE, our measured value of $T_{\mathrm{LDPE‐2},1}^{\mathrm{fus}}=379.9K$ (cf. Table 1), the value specified by the manufacturer^90^ of $T_{\mathrm{LDPE‐2},2}^{\mathrm{fus}}=389.0K$ , and the average of our measured value and that reported by the manufacturer, $T_{\mathrm{LDPE‐2},3}^{\mathrm{fus}}=384.2K$ . In Figure 7, we present SAFT-γ Mie predictions of the orthobaric $\left(P=P_{\mathrm{sol}}^{\mathrm{vap}}\right)$ solubility of LDPE-2 in toluene using these four *T* ^fus^ values and setting $\Delta h_{\mathrm{LDPE},c}^{\mathrm{fus}}=293.0J/g$ , chosen to be consistent with previous modeling of PE + solvent mixtures with SAFT-γ Mie.^79^ The ∼35 K range of values results in a very wide variation of predicted solubility values. The SAFT-γ Mie predictions are found to be in excellent agreement with our solubility data when using the manufacturer’s specified temperature of fusion, $T_{\mathrm{LDPE‐2},2}^{\mathrm{fus}}=389.0K$ ; this value is used throughout for sample LDPE-2. It is important to note that deviations in the predicted solubility when using our measured value do not necessarily indicate an inaccuracy in the measurement of $T_{\mathrm{LDPE‐2}}^{\mathrm{fus}}$ itself. The predicted solubilities also depend on the value of $\Delta h_{i}^{\mathrm{fus}}$ , and importantly, on the SAFT-γ Mie interaction parameters used to predict the activity coefficient (cf. Equation[Disp-formula eq15]), which we use in an entirely transferable manner as part of the group-contribution methodology, and which have not been refined to polymer data. It is worth noting that the use of a $T_{\mathrm{LDPE‐2}}^{\mathrm{fus}}$ value lower than 414.6 K is justified by the semicrystalline nature of the PE samples analyzed in this study. The presence of amorphous regions leads to thinner lamellar crystals and lower $T_{\mathrm{LDPE‐2}}^{\mathrm{fus}}$ values compared to a fully crystalline PE structure. This relationship is explained by the Thomson–Gibbs equation.^95^

**Figure 7:**
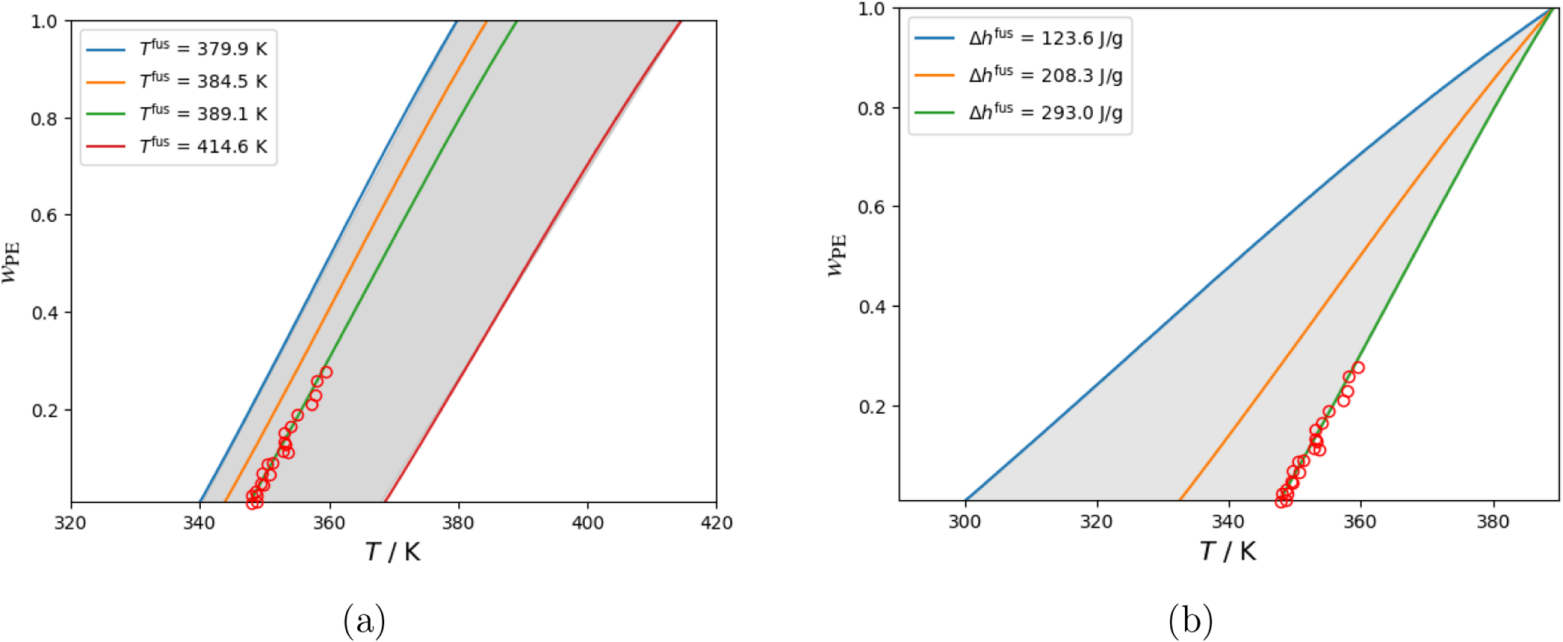
Impact of (a) the temperature of fusion and (b) the enthalpy of fusion on the calculated orthobaric SLE of LDPE-2 + toluene at a pressure equal to the solvent vapor pressure $P=P_{\mathrm{sol}}^{\mathrm{vap}}$ . The curves represent calculations with SAFT-γ Mie with $\Delta h_{\mathrm{LDPE‐2},c}^{\mathrm{fus}}=293.0J/g$ for (a) and $T_{\mathrm{LDPE‐2},2}^{\mathrm{fus}}=389.0K$ for (b). The circles represent experimental data obatined in our current work.

The impact of the value of Δ*h* ^fus^ is studied by comparing the calculated solubility of LDPE-2 in toluene using values of the enthalpy of fusion measured in the current work $\left(\Delta h_{\mathrm{LDPE}-2,1}^{\mathrm{fus}}=123.6J/g\right)$ , the reported enthalpy of fusion of fully crystalline polyethylene ( $\Delta h_{\mathrm{LDPE},c}^{\mathrm{fus}}=293.0J/g)$ , and the average of the two, $\Delta h_{\mathrm{LDPE‐2},2}^{\mathrm{fus}}=208.3J/g$ , using $T_{\mathrm{LDPE‐2},2}^{\mathrm{fus}}=389.0K$ . Although this corresponds to a very wide range of possible enthalpy of fusion values, the impact on the predicted solubility is not as pronounced as that of the variation in the temperature of fusion. As previously, we observe significantly better agreement between the SAFT-γ Mie predictions and our experimental data when the largest value of the enthalpy of fusion is used, corresponding to the fully crystalline polymer. This value of the enthalpy of fusion is chosen for all subsequent PE solubility predictions in all solvents. As discussed in the previous section, the SAFT-γ Mie parameters were transferred from smaller molecules; our investigation indicates that selecting the enthalpy of fusion of fully crystalline PE $\left(\Delta h_{\mathrm{LDPE},c}^{\mathrm{fus}}=293.0J/g\right)$ is an effective way to correct for small errors introduced by the parameter transfer and improve the accuracy of our solubility predictions, even though the PE sample is not fully crystalline.

We analyze the impact of the PE molecular weight on the PE solubility predictions in several solvents, using the same solid-state properties, $T_{\mathrm{LDPE}}^{\mathrm{fus}}=T_{\mathrm{LDPE‐2},2}^{\mathrm{fus}}=389.0K$ and $\Delta h_{\mathrm{LDPE}}^{\mathrm{fus}}=\Delta h_{\mathrm{LDPE},c}^{\mathrm{fus}}=293.0J/g$ , regardless of molecular weight. To ensure broad applicability, we carry out calculations over 1000 *M*_*w*_ values ranging from 4 to 270 kg/mol, encompassing both the PE samples studied here and other commercially relevant PEs. Our calculations in toluene and decalin, shown in Figure 8, and similar results in dibutoxymethane and dodecane (cf. Figure S1 in the SI), indicate that, overall, the molecular weight of PE has a minimal effect on solubility at a given temperature. The solubility exhibits a moderate sensitivity to molecular weight at low temperatures and at low PE molecular weights (<13 000 g/mol),[Fn fn1] although with some sensitivity also dependent on the solvent. For molecular weights greater than the (solvent-dependent) plateau, the solubility is seen to be independent of molecular weight. This suggests that polydispersity is likely not to significantly influence the calculated solubility curves of LDPE-2 (*M*_*w*_ = 105 kg/mol), but may impact the predictions for LDPE-1 (*M*_*w*_ = 4.9 kg/mol). This can be contrasted with what was observed by Paricaud et al.,^96^ who reported a strong influence of polymer molecular weight and polydispersity on SAFT (Wertheim TPT1) LLE predictions. Although they did not explicitly model polyethylene, increasing the chain length significantly broadened the LLE region of the model polymer + solvent mixture, suggesting a stronger molecular weight dependence in LLE than in SLE boundaries. This in turn led to a strong dependence of the predicted LLE behavior on the degree of polydispersity.

**Figure 8:**
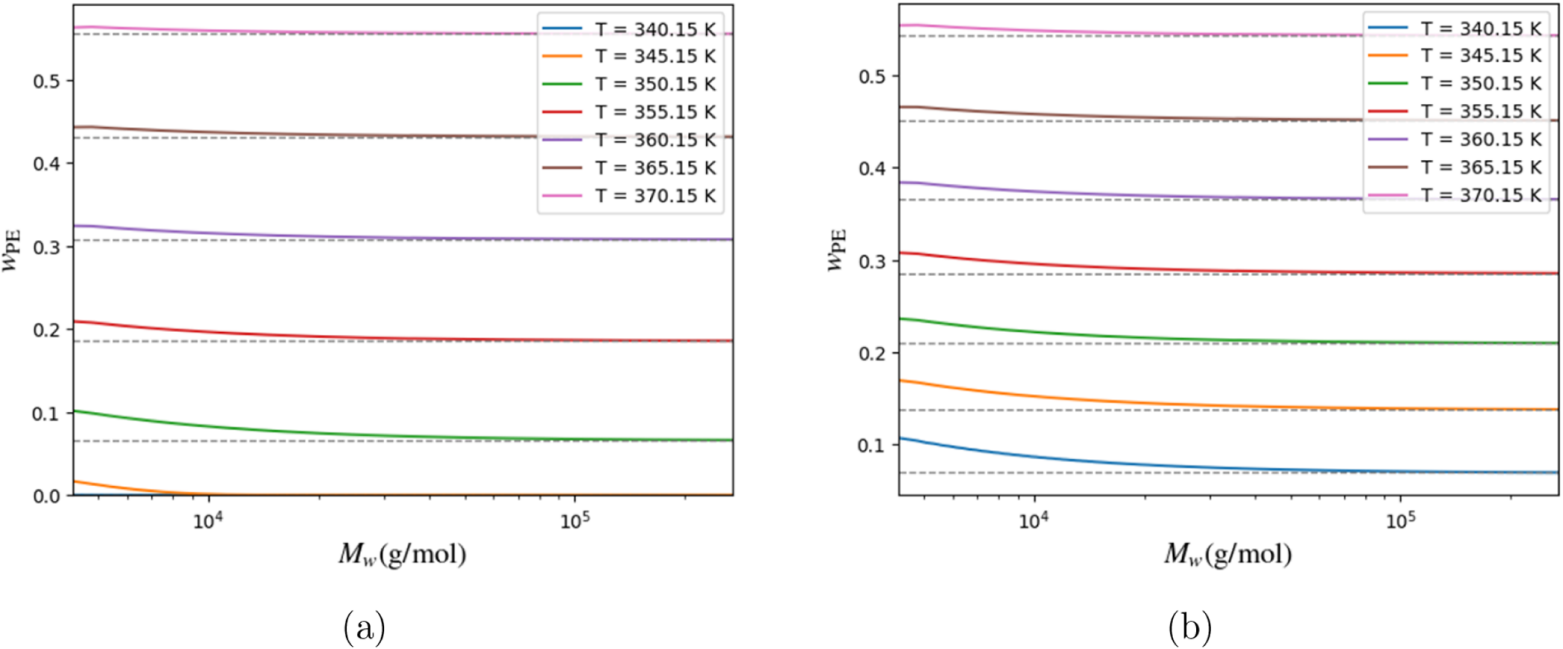
Impact of the molecular weight on the orthobaric SLE of (a) PE + toluene and (b) PE + decalin for a pressure equal to the solvent vapor pressure $P=P_{\mathrm{sol}}^{\mathrm{vap}}$ as calculated with SAFT-γ Mie. The values of $\Delta h_{\mathrm{LDPE}}^{\mathrm{fus}}=293.0J/g$ and $T_{\mathrm{LDPE}}^{\mathrm{fus}}=389.0K$ were used in the solubility calculations.

We validate further the selected value of the enthalpy of fusion by comparing SLE calculations carried out with different values of $\Delta h_{\mathrm{LDPE}}^{\mathrm{fus}}$ with SLE data from the literature.^97^ In Figure 9, we present calculations of the isobaric SLE of 17 kg/mol PE $\left(n_{{\mathrm{CH}}_{2}}=1210\right)$ + *m*-xylene at 1 atm, using *T* ^fus^ = 389.0 K^97^ and four crystalline fractions (*c*) to calculate $\Delta h_{\mathrm{LDPE}}^{\mathrm{fus}}$ through eq 17. We calculate $\Delta h_{\mathrm{LDPE}}^{\mathrm{fus}}$ as it was not measured in the experimental study.^97^ In agreement with the conclusions for the PE + toluene mixture (cf. Figure 7b), we observe that using the enthalpy of fusion of fully crystalline PE, corresponding to *c* = 1, gives the best agreement between the SAFT-γ Mie predictions and the experimental data.^97^ In the calculation of Figure 9, the experimental value of *T* ^fus^ reported in Richards et al.^97^ was used. The PE solubility calculated assuming the PE + *m*-xylene mixture behaves ideally, that is, with the activity coefficient of the polymer in solution of γ_*PE*_ = 1, is also presented in the figure for comparison and, as expected, can be seen to lead to poor agreement with the reported experimental solubilities, highlighting the highly nonideal nature of PE + solvent mixtures.

**Figure 9:**
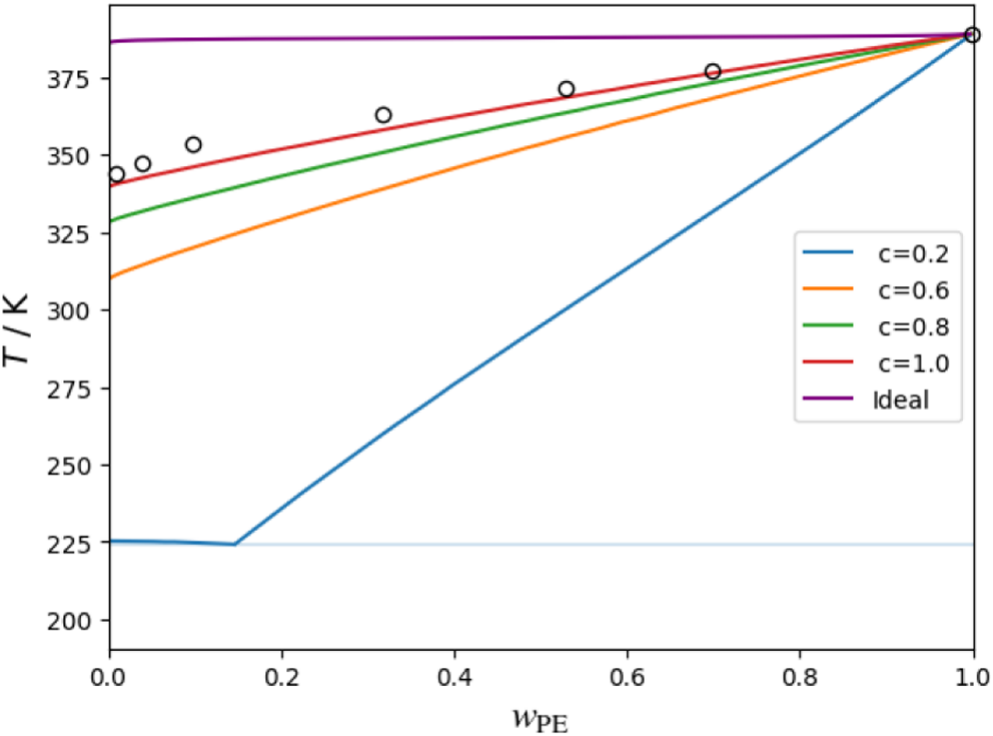
Impact of crystallinity and ideality on the isobaric SLE of 17 kg/mol polyethylene + *m*-xylene at atmospheric pressure. The purple curve is obtained by setting the activity coefficient of polyethylene in the solution to 1, and the other curves represent calculations with the activity coefficient as predicted by SAFT-γ Mie, using *T* ^fus^ = 389.0 K^97^ and crystallinity-dependent values of the enthalpy of fusion. For *m*-xylene values of *T* ^fus^ = 225.30 K and Δ*h* ^fus^ = 109.27 J/g were used.^91^ The circles represent experimental data from Richards et al.^97^

### Modeling of Selected PE + Solvent Mixtures with SAFT-γ Mie

We now carry out predictive SAFT-γ Mie calculations of the SLE and solid–liquid–liquid equilibrium (SLLE) of PE + *n*-alkanes, cyclic solvents, aromatic solvents, and two green solvents. These mixtures and types of phase behavior are relevant to the experimental conditions explored in the current study and to the plastic recycling process we aim to address. Additionally, we analyze the dependence of solubility curves on properties of the PE samples. We use the following solid-state properties for all calculations: $\Delta h_{\mathrm{LDPE‐1}}^{\mathrm{fus}}=293.0J/g$ and $T_{\mathrm{LDPE‐1}}^{\mathrm{fus}}=375.2K$ , as measured in the current work, for LDPE-1; $\Delta h_{\mathrm{LDPE‐2}}^{\mathrm{fus}}=293.0J/g$ and $T_{\mathrm{LDPE‐2}}^{\mathrm{fus}}=389.0K$ , specified by the manufacturer, for LDPE-2.

In Figure 10, we present the SLE of LDPE-1 and LDPE-2 in *n*-dodecane, and compare it with measured solubilities. This comparison involves using the CH_3_–CH_2_ interaction parameters^73^ in the context of PE + *n*-alkane mixtures. Given that the enthalpy and temperature of fusion of LDPE-2 are higher than those of LDPE-1 (cf. Table 1), we find that the predicted SLE temperature of LDPE-2 is higher than that of LDPE-1 at all *w*_PE_, in agreement with the experimental data. Close agreement is observed between the SAFT-γ Mie predictions and our experimental measurements and for LDPE-1, while a poorer agreement is found for LDPE-2.

**Figure 10:**
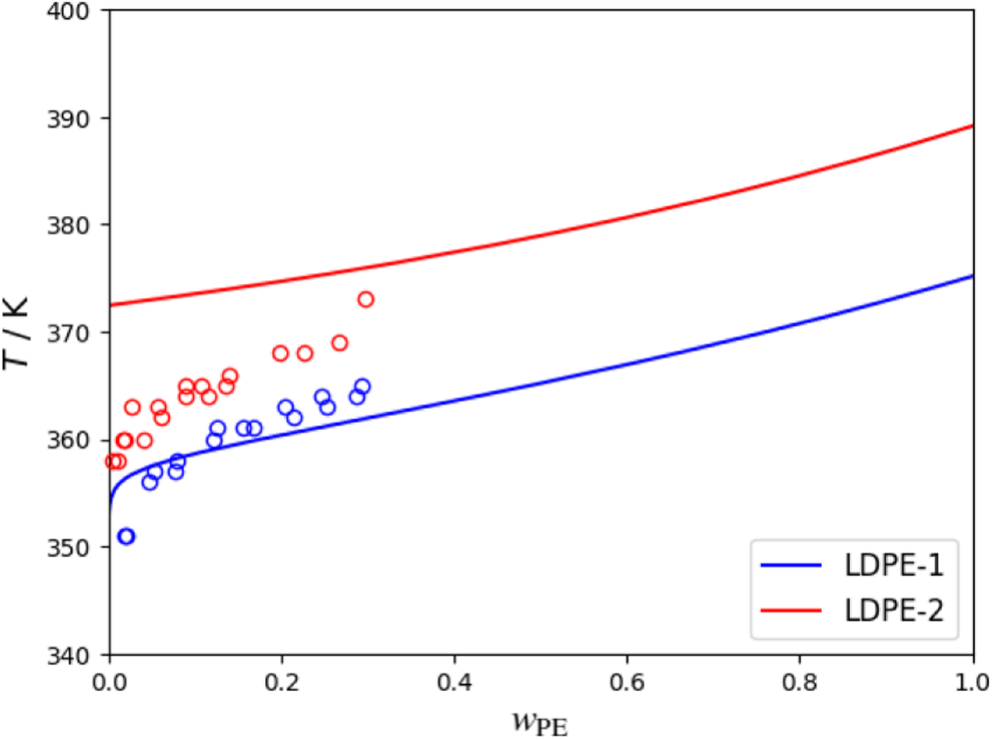
Orthobaric SLE of LDPE-1 and LDPE-2 in *n*-dodecane at the solvent vapor pressure $P=P_{\mathrm{sol}}^{\mathrm{vap}}$ . The curves represent predictions with SAFT-γ Mie with $\Delta h_{\mathrm{LDPE‐1}}^{\mathrm{fus}}=\Delta h_{\mathrm{LDPE‐2}}^{\mathrm{fus}}$ = 293.0 J/g, $T_{\mathrm{LDPE‐1}}^{\mathrm{fus}}$ = 375.2 K and $T_{\mathrm{LDPE‐2}}^{\mathrm{fus}}$ = 389.0 K. The circles represent experimental data from the current work.

In the case of mixtures involving a cyclic solvent, namely decalin or cyclohexanone, calculations require the CH_2_–cCH,^74^ CH_2_–cCH_2_,^74^ and CH_2_–cCO^88^ interaction parameters in the context of PE + solvent mixtures. The orthobaric SLE at the solvent vapor pressure for LDPE-1 and LDPE-2 in decalin is presented in Figure 11a. As expected, a higher solubility temperature is predicted for LDPE-2. Close agreement is observed between the SAFT-γ Mie calculations and the experimental data for the LDPE-2 sample. Interestingly, poorer agreement is found for LDPE-1. This deviation from the experimental data most likely arises from the influence of polydispersity on the LDPE-1 sample due to its low *M*_*w*_.

**Figure 11:**
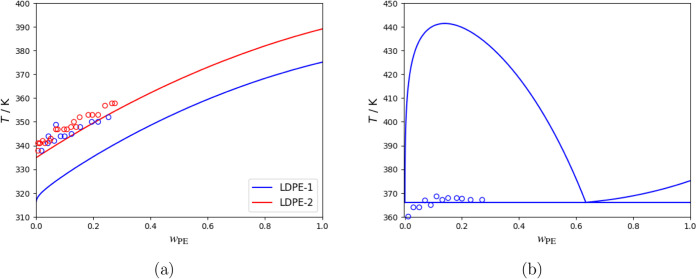
Phase diagrams of LDPE in cyclic solvents. (a) Independent orthobaric SLE curves for pure LDPE-1 in decalin and pure LDPE-2 in decalin, plotted on the same axes at $P=P_{\mathrm{sol}}^{\mathrm{vap}}$ (these are separate systems, not a polymer blend). The *w*_PE_ axis represents the weight fraction of the respective pure polymer. (b) Orthobaric SLLE of the LDPE-1 + cyclohexanone system at $P=P_{\mathrm{sol}}^{\mathrm{vap}}$ . The curves represent calculations with SAFT-γ Mie using $\Delta h_{\mathrm{LDPE‐1}}^{\mathrm{fus}}=\Delta h_{\mathrm{LDPE‐2}}^{\mathrm{fus}}=293.0J/g$ , $T_{\mathrm{LDPE‐1}}^{\mathrm{fus}}=375.2K$ , and $T_{\mathrm{LDPE‐2}}^{\mathrm{fus}}=389.0K$ . The symbols represent independent experimental data from our current work.

In Figure 11b, the phase diagram of the LDPE-1 + cyclohexanone mixture at the solvent vapor pressure is presented. Three-phase equilibrium (SLLE) is observed at 366.2 K and the SAFT-γ Mie predictions for this mixture are in good agreement with our measured data. However, the model underpredicts the weight fraction of LDPE-1 on the solvent-rich side of the LLE envelope. SAFT-γ Mie predicts an upper critical solution temperature (UCST) at approximately 440 K. This result suggests that polar solvents, such as cyclohexanone, can lead to liquid–liquid demixing when used to dissolve PE and may be less suitable for dissolving a polyolefin like PE than nonpolar solvents, such as *n*-dodecane or decalin, which are more chemically similar to the polymer.

In Figure 12, the orthobaric SLE for LDPE-1 and LDPE-2 in three aromatic solvents, toluene, *p*-xylene, and mesitylene, at the solvent vapor pressure, is presented. These calculations require the CH_2_–aCH and CH_2_–aCCH_3_ ^74^ interaction parameters in the context of PE + solvent mixtures. In Figure 12a, where the SLE of LDPE-1 and LDPE-2 in toluene is shown, an excellent match between the SAFT-γ Mie predictions and our experimental data can be seen. A close correspondence is also evident in Figure 12b, in which we show the SLE of LDPE-1 and LDPE-2 + *p*-xylene. Larger deviations are observed in Figure 12c for LDPE-1 and LDPE-2 + mesitylene mixtures, although qualitative agreement is maintained.

**Figure 12:**
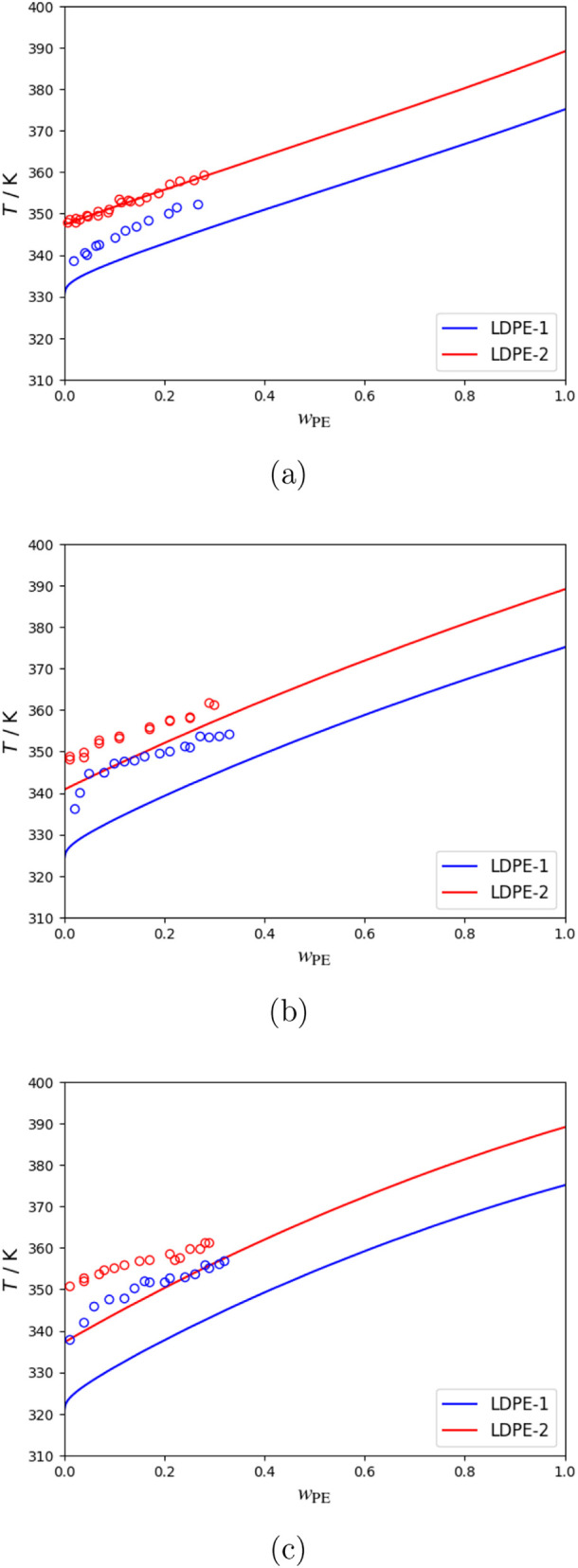
Orthobaric SLE of LDPE-1 and LDPE-2 in three aromatic solvents at the solvent vapor pressure $P=P_{\mathrm{sol}}^{\mathrm{vap}}$ : (a) toluene, (b) *p*-xylene and (c) mesitylene. The curves represent calculations with SAFT-γ Mie using $\Delta h_{\mathrm{LDPE‐1}}^{\mathrm{fus}}=\Delta h_{\mathrm{LDPE‐2}}^{\mathrm{fus}}$ = 293.0 J/g, $T_{\mathrm{LDPE‐1}}^{\mathrm{fus}}$ = 375.2 K and $T_{\mathrm{LDPE‐2}}^{\mathrm{fus}}$ = 389.0 K. The circles represent experimental data from this work.

In Figure 13, the orthobaric SLE are presented for LDPE-1 and LDPE-2 in two green solvents, *p*-cymene and dibutoxymethane, at the solvent vapor pressure. The calculations require the CH_2_–aCCH^74^ and CH_2_–mO^98^ interaction parameters in the context of PE + solvent mixtures. In Figure 13, close agreement is found between the SAFT-γ Mie predictions and our experimental data for LDPE-1 in *p*-cymene and LDPE-2 in dibutoxymethane.

**Figure 13:**
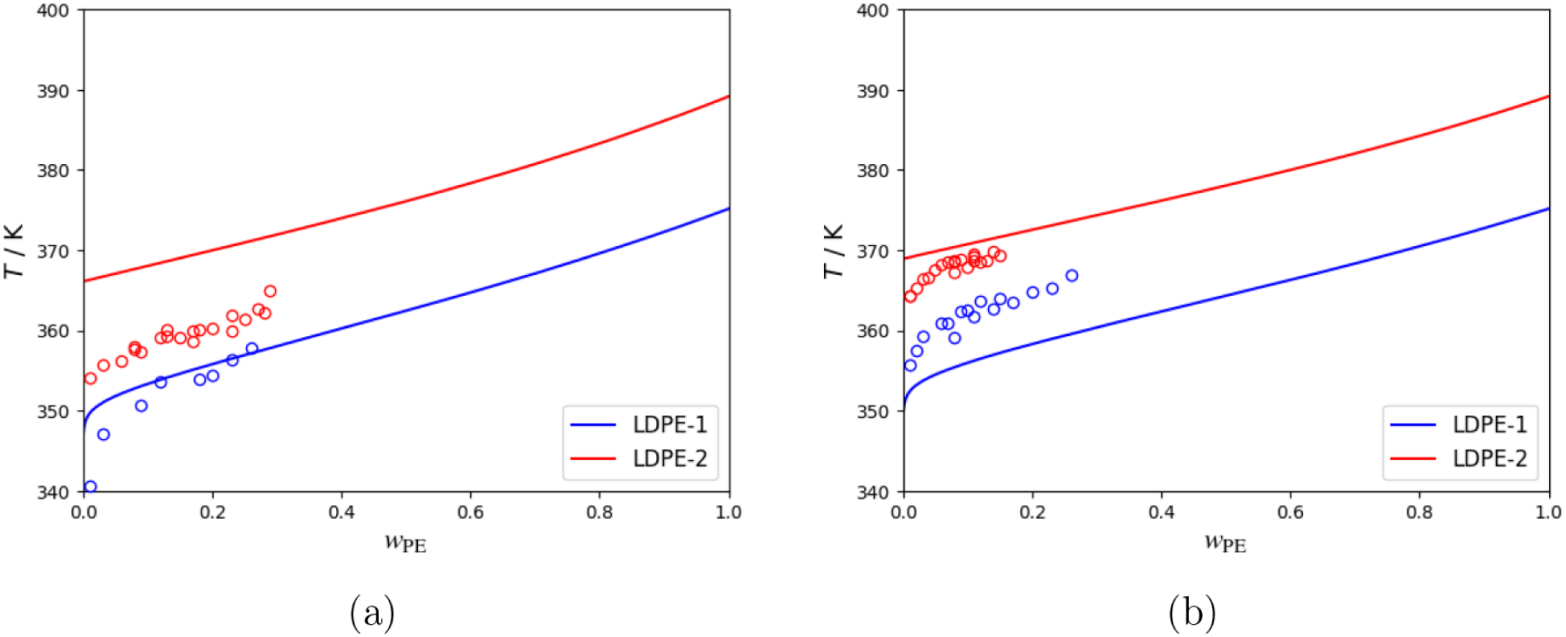
Orthobaric SLE of LDPE-1 and LDPE-2 in two green solvents, at the solvent vapor pressure $P=P_{\mathrm{sol}}^{\mathrm{vap}}$ : (a) *p*-cymene and (b) dibutoxymethane. The curves represent calculations with SAFT-γ Mie using $\Delta h_{\mathrm{LDPE‐1}}^{\mathrm{fus}}=\Delta h_{\mathrm{LDPE‐2}}^{\mathrm{fus}}=293.0\;J/g$ , $T_{\mathrm{LDPE‐1}}^{\mathrm{fus}}=375.2\;K$ , and $T_{\mathrm{LDPE‐2}}^{\mathrm{fus}}=389.0\;K$ . The circles represent experimental data from our current work.

In Figure 14, we compare the experimental solubilities of LDPE-1 and LDPE-2 in toluene, decalin, *n*-dodecane, dibutoxymethane, *p*-cymene, mesitylene, and *p*-xylene at the solvent vapor pressure with SAFT-γ Mie calculations. The accurate prediction of solvent rankings based on polymer solubility is essential for integrating SAFT-γ Mie into a solvent and process design framework, with the goal of optimizing polymer recycling via dissolution/precipitation.

**Figure 14:**
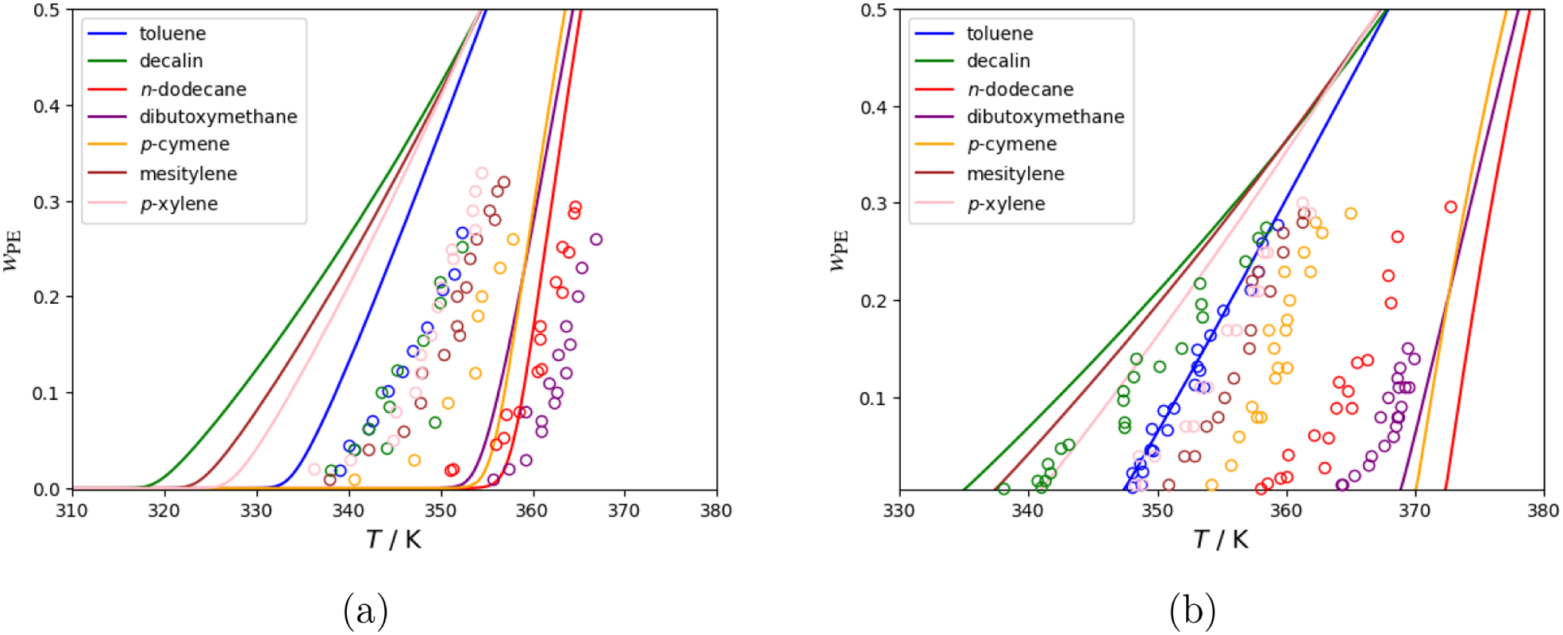
Solubility, in mass fraction, of two polymer samples in toluene, decalin, *n*-dodecane, dibutoxymethane, *p*-cymene, mesitylene, and *p*-xylene, at the solvent vapor pressure $P=P_{\mathrm{sol}}^{\mathrm{vap}}$ : (a) LDPE-1, and (b) LDPE-2. The curves represent solubility calculations with SAFT-γ Mie using $\Delta h_{\mathrm{LDPE‐1}}^{\mathrm{fus}}=\Delta h_{\mathrm{LDPE‐2}}^{\mathrm{fus}}=293.0\;J/g$ , $T_{\mathrm{LDPE‐1}}^{\mathrm{fus}}=375.2\;K$ , and $T_{\mathrm{LDPE‐2}}^{\mathrm{fus}}=389.0\;K$ . The circles represent experimental data from our current work.

For a given solubility *w*_PE_, the solvent rankings based on SLE temperature (from lowest to highest temperature, corresponding to highest to lowest solubility) are summarized in Table 3. According to SAFT-γ Mie, the predicted order is identical for both LDPE-1 and LDPE-2, starting with decalin as the best solvent and ending with *n*-dodecane. Notably, the model predicts that the curves for dibutoxymethane and *p*-cymene are nearly overlapping.

**Table 3:** Comparison of Solvent Rankings Based on Hansen Solubility Parameters (HSP RED), Experimental Dissolution Temperatures, and SAFT-*γ* Mie Predictions for LDPE-1 and LDPE-2^a^

		LDPE-1 Rank		LDPE-2 Rank	
Solvent	HSP (RED) Rank	Exp	SAFT-γ Mie	Exp	SAFT-γ Mie
Toluene	5	1	4	2	4
Decalin	7	2	1	1	1
*p*-Xylene	4	3	3	3	3
Mesitylene	6	4	2	4	2
*p*-Cymene	2	5	5	5	5
*n*-Dodecane	3	6	7	6	7
Dibutoxymethane	1	7	6	7	6

a Solvents are ordered by their experimental ranking for LDPE-1. (For HSP, Rank 1 indicates the lowest RED. For experimental and SAFT-*γ* Mie, Rank 1 indicates the lowest required dissolution temperature at a given polymer weight fraction).

Experimentally, as shown in Table 3, the ranking for LDPE-1 is led by toluene, decalin, and *p*-xylene, with these first three solvents exhibiting nearly overlapping behavior. For LDPE-2, the experimental order shifts slightly, with decalin appearing as the most effective solvent followed by toluene. In contrast to the model’s prediction where *n*-dodecane is ranked lowest, the experimental data consistently places dibutoxymethane as the poorest solvent for both polymers. More broadly, there is agreement between the calculations and experimental data that decalin, mesitylene, *p*-xylene and toluene are generally ″good″ solvents while dibutoxymethane and *n*-dodecane are ″bad″ solvents. This level of agreement is deemed sufficient to support the identification of promising solvent candidates during process development.

More broadly, the qualitative HSP RED rankings exhibit significant deviations from the experimentally obtained SLE temperatures. For instance, decalin possesses the highest RED value among the final candidates—suggesting the lowest solubility—yet it experimentally exhibits the lowest required dissolution temperatures for both polymers. Conversely, dibutoxymethane yields the lowest RED value but requires the highest experimental temperatures for dissolution. Overall, the SAFT-γ Mie calculations demonstrate good agreement with the experimental SLE data, identifying decalin, mesitylene, *p*-xylene, and toluene as highly effective solvents (requiring lower dissolution temperatures), while classifying dibutoxymethane and *n*-dodecane as less effective solvents.

### Accuracy of the Model

In Figure 15, we present parity plots with the predicted and the experimental SLE temperatures for LDPE-1 and LDPE-2 in the solvents studied at the same *w*_PE_. As can be seen, the SAFT-γ Mie calculations are found to be within 20 K of the measurements, a satisfactory level of agreement in the context of fully predictive thermodynamic models, given the uncertainty in the solid-state properties of the polymer samples.^99^ It is noteworthy that the agreement is best for LDPE-2, the PE sample with a higher temperature of fusion and molecular weight, for all solvents except *n*-dodecane. As the SAFT-γ Mie group interaction parameters are fitted to small molecule fluid-phase behavior, one may have expected larger deviations for larger polymers (which contain more repetitions of given groups; e.g., CH_2_ here). We find this, however, not to be the case. As discussed previously, the solubility predictions for lower molecular weight LDPE-1 are likely influenced by the sample’s polydispersity. Furthermore, low-density PE is known to exhibit significant branching,^54^ but this factor has not been explored in our work. An experimental investigation of the degree of branching and the development of polymer-specific groups to account for branching may enhance the agreement with the experimental data further.

**Figure 15:**
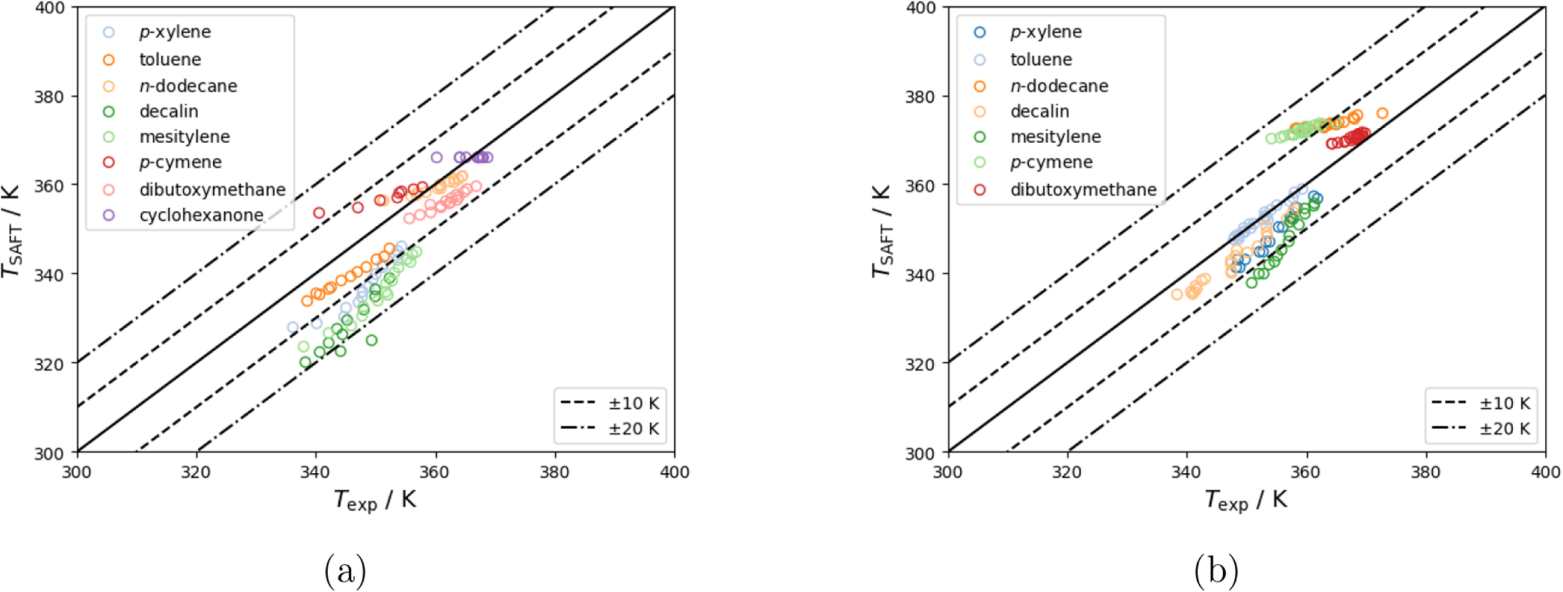
Parity plot of the SLE temperatures calculated with SAFT-γ Mie vs experimentally measured SLE temperatures at the same polymer mass fraction *w*_PE_ for two polymer samples in a range of solvents and at $P=P_{\mathrm{sol}}^{\mathrm{vap}}$ : (a) LDPE-1 and (b) LDPE-2. The values of $\Delta h_{\mathrm{LDPE‐1}}^{\mathrm{fus}}=\Delta h_{\mathrm{LDPE‐2}}^{\mathrm{fus}}=293.0\;J/g$ , $T_{\mathrm{LDPE‐1}}^{\mathrm{fus}}=375.2\;K$ , and $T_{\mathrm{LDPE‐2}}^{\mathrm{fus}}=389.0\;K$ are used in the calculations.

We provide a quantitative analysis of the accuracy of the SAFT-γ Mie predictions in Table 4, reporting the %AAD and AAD of the equilibrium temperature for each PE (LDPE-1, MDPE, LDPE-2, LDPE-3 and 17 kg/mol PE) + solvent combination considered in this section (cf. eqs 19 and 20, respectively). These deviations were evaluated using the *T*_SLE_ and, in the case of LDPE-1 + cyclohexanone, the *T*_SLLE_ predicted with SAFT-γ Mie, with the corresponding experimental weight fraction employed as input to the calculations. For each system and property, we indicate the number of experimental points used in the evaluation together with the thermodynamic conditions employed. The largest deviations observed are 5.17% and 18.4 K for the MDPE + mesitylene system. This result represents excellent accuracy for a group-contribution method that does not involve fitting to polymer experimental data. Importantly, the deviation obtained is comparable to the uncertainty in the temperature of fusion itself: for example, we measured *T* ^fus^ values of 375.2 and 379.9 K for LDPE-1 and LDPE-2, while the manufacturer reports 389.0 K for LDPE-2, and the melting point of fully crystalline PE is 414.6 K. A broader assessment of the model’s performance, detailing the distribution of average errors and standard deviations across all samples, is available in the Supporting Information (Figure S10).

**Table 4:** Overview of the Accuracy of SAFT-*γ* Mie in the Calculation of SLE or SLLE Temperature of PE + Solvent Mixtures^a^ ^b^

System *s*								
PE sample	Solvent	*T*	*P*/kPa	*w*_PE_	$N_{s,p}^{\mathrm{tot}}$	%AAD	AAD/K	ref.
LDPE-1	*p*-cymene	*T*_SLE_	$P=P_{\mathrm{sol}}^{\mathrm{vap}}$	0.0100–0.260	8	1.50	5.23	*
	decalin	*T*_SLE_	$P=P_{\mathrm{sol}}^{\mathrm{vap}}$	0.0193–0.252	12	5.02	17.35	*
	*n*-dodecane	*T*_SLE_	$P=P_{\mathrm{sol}}^{\mathrm{vap}}$	0.0193–0.294	16	0.582	2.09	*
	dibutoxymethane	*T*_SLE_	$P=P_{\mathrm{sol}}^{\mathrm{vap}}$	0.0100–0.260	16	1.62	5.85	*
	toluene	*T*_SLE_	$P=P_{\mathrm{sol}}^{\mathrm{vap}}$	0.0193–0.267	12	1.77	6.12	*
	mesitylene	*T*_SLE_	$P=P_{\mathrm{sol}}^{\mathrm{vap}}$	0.0100–0.320	16	4.15	14.5	*
	*p*-xylene	*T*_SLE_	$P=P_{\mathrm{sol}}^{\mathrm{vap}}$	0.0200–0.330	16	3.13	10.9	*
	cyclohexanone	*T*_SLLE_	$P=P_{\mathrm{sol}}^{\mathrm{vap}}$	0.0100–0.270	12	0.545	1.99	*
LDPE-2	*p*-cymene	*T*_SLE_	$P=P_{\mathrm{sol}}^{\mathrm{vap}}$	0.01–0.29	20	3.52	12.63	*
	decalin	*T*_SLE_	$P=P_{\mathrm{sol}}^{\mathrm{vap}}$	0.00601–0.275	22	1.35	4.69	*
	*n*-dodecane	*T*_SLE_	$P=P_{\mathrm{sol}}^{\mathrm{vap}}$	0.00546–0.297	18	2.66	9.64	*
	dibutoxymethane	*T*_SLE_	$P=P_{\mathrm{sol}}^{\mathrm{vap}}$	0.0100–0.150	20	0.700	2.57	*
	toluene	*T*_SLE_	$P=P_{\mathrm{sol}}^{\mathrm{vap}}$	0.00738–0.278	22	0.138	0.458	*
	mesitylene	*T*_SLE_	$P=P_{\mathrm{sol}}^{\mathrm{vap}}$	0.0100–0.290	16	2.54	9.04	*
	*p*-xylene	*T*_SLE_	$P=P_{\mathrm{sol}}^{\mathrm{vap}}$	0.0100–0.300	16	1.61	5.71	*
MDPE	*p*-cymene	*T*_SLE_	$P=P_{\mathrm{sol}}^{\mathrm{vap}}$	0.00879–0.289	17	0.57	2.03	*
	decalin	*T*_SLE_	$P=P_{\mathrm{sol}}^{\mathrm{vap}}$	0.0178–0.251	12	4.81	16.94	*
	*n*-dodecane	*T*_SLE_	$P=P_{\mathrm{sol}}^{\mathrm{vap}}$	0.0177–0.297	16	0.871	3.19	*
	dibutoxymethane	*T*_SLE_	$P=P_{\mathrm{sol}}^{\mathrm{vap}}$	0.00486–0.151	14	1.77	6.53	*
	toluene	*T*_SLE_	$P=P_{\mathrm{sol}}^{\mathrm{vap}}$	0.0196–0.261	11	2.65	9.36	*
	mesitylene	*T*_SLE_	$P=P_{\mathrm{sol}}^{\mathrm{vap}}$	0.0141–0.289	15	5.17	18.4	*
	*p*-xylene	*T*_SLE_	$P=P_{\mathrm{sol}}^{\mathrm{vap}}$	0.0134–0.298	15	4.25	15.05	*
	cyclohexanone	*T*_SLLE_	$P=P_{\mathrm{sol}}^{\mathrm{vap}}$	0.0114–0.258	9	1.67	4.34	*
LDPE-3	*p*-cymene	*T*_SLE_	$P=P_{\mathrm{sol}}^{\mathrm{vap}}$	0.0128–0.354	8	1.05	3.96	*
	decalin	*T*_SLE_	$P=P_{\mathrm{sol}}^{\mathrm{vap}}$	0.00583–0.344	7	4.06	15.01	*
	*n*-dodecane	*T*_SLE_	$P=P_{\mathrm{sol}}^{\mathrm{vap}}$	0.0322–0.353	7	0.362	1.38	*
	dibutoxymethane	*T*_SLE_	$P=P_{\mathrm{sol}}^{\mathrm{vap}}$	0.0122–0.328	8	0.86	3.30	*
	toluene	*T*_SLE_	$P=P_{\mathrm{sol}}^{\mathrm{vap}}$	0.00686–0.253	9	2.28	8.42	*
	mesitylene	*T*_SLE_	$P=P_{\mathrm{sol}}^{\mathrm{vap}}$	0.0114–0.350	8	4.53	16.90	*
	*p*-xylene	*T*_SLE_	$P=P_{\mathrm{sol}}^{\mathrm{vap}}$	0.00965–0.351	8	3.68	13.71	*
	cyclohexanone	*T*_SLLE_	$P=P_{\mathrm{sol}}^{\mathrm{vap}}$	0.0108–0.331	7	0.212	0.821	*
PE 17 kg/mol	*m*-xylene	*T*_SLE_	101.325	0.00838–0.698	6	1.23	4.39	^97^

a The first two columns specify the system *S* and the third column the property *p* being compared to experimental data (the SLE temperature *T*_SLE_ or the SLLE temperature *T*_SLLE_), *P* is the pressure of the calculation/experiment, *w*_PE_ the range of weight fractions of the polymer, $N_{s,p}^{\mathrm{tot}}$ the total number of experimental data points used to calculate %AAD*_s,p_* and AAD*_s,p_*, for system *S* and property *p*

b * Denotes current work

## Conclusions

We have conducted a systematic investigation of the temperature dependence of polyethylene solubility in various solvents, including both conventional and bioderived candidates featuring different functional groups. From an initial pool of 61 solvents, 33 were shortlisted based on low RED values, as derived from Hansen solubility parameter calculations, and further refined by considering their normal boiling points and toxicity. A preliminary screening at a PE weight fraction of 0.01 g/g identified 10 solvents capable of dissolving PE: decalin, toluene, *p*-xylene, limonene, *p*-cymene, α-pinene, mesitylene, *n*-dodecane, cyclohexanone, and dibutoxymethane.

We performed solubility measurements for four PE samples differing in molecular weight, density, and solid-state properties (temperature and specific enthalpy of fusion) in the 10 selected solvents. Our results show that melting properties—specifically temperature and enthalpy of fusion, which are related to the crystallinity of the PE sample—dominate solubility, outweighing the effects of molecular weight and density in low to medium polymer densities. This is exemplified by MDPE and LDPE-2, which, despite differing in *M*_*w*_ by more than an order of magnitude and differing densities, exhibit nearly identical solubility profiles due to their similar melting properties. Notably, the ranking of solvent + PE SLE temperatures remains largely consistent across PE grades, with the traditional solvents decalin, toluene, and *p*-xylene outperforming the other solvents. Encouragingly, the bioderived solvents limonene, *p*-cymene, and α-pinene, were found to exhibit comparable SLE temperatures to these traditional solvents, offering promising potential as sustainably sourced alternatives.

We carried out fully predictive SAFT-γ Mie calculations of the SLE of PE + *n*-dodecane, decalin, toluene, *p-*xylene, *m*-xylene, mesitylene, *p*-cymene, and dibutoxymethane binary mixtures as well as for the SLLE of PE + cyclohexanone binary mixtures. We observed that the melting properties – enthalpy and temperature of fusion – have a significant impact on the SLE predictions, consistent with experimental observations. We found that using the PE sample-specific temperature of fusion and the enthalpy of fusion of fully crystalline PE for the SAFT-γ Mie calculations offers the best agreement with experimental data. Furthermore, we observed that the polymer molecular weight affects solubility predictions only at low *M*_*w*_ values and temperatures. This work has allowed us to assess the transferability of the existing database of SAFT-γ Mie parameters, relevant to *n*-alkanes, ethers, cyclic, and aromatic solvents, using both our experimental data and data from the literature. Across all systems, the model delivers satisfactory accuracy, with %AAD and AAD not exceeding 5.17% and 18.4 K (observed for MDPE + mesitylene), respectively. Parity plots and PE + solvent phase diagrams confirmed the reliability of the SAFT-γ Mie group-contribution approach, which has been applied here in a fully predictive manner, providing a robust foundation for integrated molecular and process design frameworks. In practice, this predictive framework can provide key thermodynamic inputs for process simulation, enabling the design of plastic recycling processes, even for mixed waste streams of unknown composition, by evaluating a range of polymer properties.^100^ Further refinement of group interactions with polymer data and accounting for branching in LDPE may improve the accuracy of the model.

These findings offer practical insights for optimizing the solvent-based recycling, contributing to more sustainable PE recycling practices. Future research could focus on extending the solvent range to additional environmentally benign solvents, exploring other polyolefin types, such as polypropylene (PP) and high-density polyethylene (HDPE), potentially mixed with common additives. This would allow for further investigation of the thermodynamics of solvent-based recycling, especially for complex waste streams such as multilayer and contaminated plastics.^101,102^

## Supplementary Material



## Data Availability

Data underlying this article can be accessed on Zenodo at 10.5281/zenodo.20029538 and used under the Creative Commons Attribution license.

## Footnotes

1 The *M*_*w*_ corresponding to the onset of the solubility plateau was identified as the molecular weight at which the absolute slope of *w*_PE_ vs *M*_*w*_, estimated via finite differences, remained below a threshold (*ε* = 10^–6^) over ten consecutive points for a given *T* and solvent. This *M*_*w*_ value was then averaged across temperatures and solvents. Further details of the methodology are included in the SI (cf. Equations (S1), (S2)).

## Notes

Hansen solubility parameters and solvent screening criteria; experimentally measured PE solubility data; SAFT-γ Mie group parameter table; analysis of the influence of PE molecular weight on solubility predictions using Schulz–Flory distributions; modeling of selected polyethylene-solvent mixtures with SAFT-γ Mie; and absolute average deviation distributions across PE samples (PDF)
