# Supplementary material for: Investigating Polyethylene Solubility for Solvent-Based Recycling: Experiments and SAFT‑γ Mie Predictions
Source: Macromolecules. 2026 Jun 8;59(12):7011–30. doi: 10.1021/acs.macromol.6c00489 (PMC13296513; doi:10.1021/acs.macromol.6c00489)
Supplement: Supplementary file 1 [file ma6c00489_si_001.pdf]

Investigating Polyethylene Solubility for Solvent-Based Recycling: Experiments and SAFT-γ Mie Predictions (Supporting Information) †

Riccardo Standish [‖,‡] Jian Yin [‖,¶] Jakob Burger,[§] George Jackson,[‡] Claire S. Adjiman,[‡] Mirjana Minceva,[*,¶] and Amparo Galindo[*,‡]

‡*Department of Chemical Engineering, Sargent Centre for Process Systems Engineering, Imperial College London, London SW7 2AZ, UK*

¶*TUM School of Life Sciences, Technical University of Munich, Germany*

§*Campus Straubing for Biotechnology and Sustainability, Technical University of Munich, Germany*

E-mail: mirjana.minceva@tum.de; a.galindo@imperial.ac.uk

[†] *Corresponding authors. *Email addresses*: a.galindo@imperial.ac.uk (Amparo Galindo), mirjana.minceva@tum.de (Mirjana Minceva). [‖] These authors contributed equally to this work.

Solvent selection

The thresholds for this initial screening in Table S1 were established strictly to safely facilitate laboratory-scale equilibrium measurements rather than to optimize industrial process metrics. For Criterion 2, a minimum boiling point threshold of 338 K (approximately the boiling point of THF) was selected; highly volatile solvents below this threshold pose equipment safety risks during prolonged heating at the required test conditions (10 K below the boiling point). For Criterion 4, the 24-hour dissolution requirement was implemented as a conservative laboratory screening window rather than a target industrial residence time, ensuring that solvents thermodynamically capable of dissolving PE were not prematurely rejected due to slow dissolution kinetics.

Table S1: Hansen solubility parameters and screening criteria for solvents. RED is the Relative Energy Difference (R_a/R_0). The final column indicates the screening outcome and the reason for rejection, such as failing Criterion 1 (C1: RED $>$ 1), failing Criterion 2 (C2: boiling point $<$ 383 K), failing Criterion 3 (C3: commonly known hazardous solvent), or failing Criterion 4 (C4: incomplete dissolution after 24 hours).

**Solvent**	δ_d (MPa$^{1/2}$)	δ_p (MPa$^{1/2}$)	δ_h (MPa$^{1/2}$)	RED	**Outcome & Reason**
Dibutoxymethane	15.70	4.00	3.90	0.32	Accepted
Diethyl Sulfide	16.80	3.10	2.00	0.32	Rejected (Fails C3)
p-cymene	17.30	2.40	2.40	0.48	Accepted
α-Pinene	16.90	1.80	3.10	0.50	Accepted
CO_2	15.60	5.20	5.80	0.54	Rejected (Fails C2)
Dimethyl Ether	15.20	6.10	5.70	0.57	Rejected (Fails C2)
Diethyl Ether	14.50	2.90	4.60	0.58	Rejected (Fails C2)
D-limonene	17.20	1.80	4.30	0.62	Accepted
Tetrachloroethylene	18.30	5.70	0.00	0.64	Rejected (Fails C3)
dodecane	16.00	0.00	0.00	0.64	Accepted
Dihydropyran	17.50	5.50	5.70	0.66	Rejected (Fails C4)
Furan	17.00	1.80	5.30	0.67	Rejected (Fails C2)
Cyclohexane	16.80	0.00	0.20	0.67	Rejected (Fails C4)
MEK (Methyl Ethyl Ketone)	16.00	9.00	5.00	0.67	Rejected (Fails C4)
p-xylene	17.60	1.00	3.10	0.68	Accepted
Cyclohexanone	17.80	6.30	5.10	0.68	Accepted

Table S1: continued

**Solvent**	δ_d (MPa$^{1/2}$)	δ_p (MPa$^{1/2}$)	δ_h (MPa$^{1/2}$)	RED	**Outcome & Reason**
Toluene	18.00	1.40	2.00	0.69	Accepted
Hexane	14.90	0.00	0.00	0.69	Rejected (Fails C4)
Ethyl acetate	15.80	5.30	7.20	0.71	Rejected (Fails C4)
Pentane	14.50	0.00	0.00	0.73	Rejected (Fails C2)
Chloroform	17.80	3.10	5.70	0.74	Rejected (Fails C2)
Mesitylene	18.00	0.60	0.60	0.76	Accepted
Chlorobenzene	19.00	4.30	2.00	0.78	Rejected (Fails C3)
Butane	14.10	0.00	0.00	0.78	Rejected (Fails C2)
Carbon tetrachloride	17.80	0.00	0.60	0.78	Rejected (Fails C3)
Decalin	18.00	0.00	0.00	0.83	Accepted
DCM (dichloromethane)	18.20	6.30	6.10	0.83	Rejected (Fails C2)
m-Dichlorobenzene	19.20	5.10	2.70	0.84	Rejected (Fails C3)
THF (tetrahydrofuran)	16.80	5.70	8.00	0.85	Rejected (Fails C4)
Benzene	18.40	0.00	2.00	0.88	Rejected (Fails C3)
Bromobenzene	19.20	5.50	4.10	0.89	Rejected (Fails C3)
propane	13.40	0.00	0.00	0.89	Rejected (Fails C2)
Acetone	15.50	10.40	7.00	0.98	Rejected (Fails C2)
γ-Valerolactone	16.90	11.50	6.30	1.05	Rejected (Fails C1)
1-Decanol	16.00	4.70	10.50	1.13	Rejected (Fails C1)
1,4 dioxane	19.00	1.80	7.40	1.14	Rejected (Fails C1)
Tetradecanol	18.34	2.21	9.41	1.21	Rejected (Fails C1)
Benzoic acid	18.20	6.90	9.80	1.21	Rejected (Fails C1)
Cyrene	18.80	10.60	6.90	1.22	Rejected (Fails C1)
Carbondisulfide	20.20	0.00	0.60	1.24	Rejected (Fails C1)
NMP	18.00	12.30	7.20	1.28	Rejected (Fails C1)
Borneol	14.55	3.28	11.45	1.30	Rejected (Fails C1)
DMAc (Dimethylacetamide)	16.80	11.50	10.20	1.38	Rejected (Fails C1)
Thymol	19.00	4.50	10.80	1.40	Rejected (Fails C1)
Isoamyl Alcohol (3-Methyl-1-Butanol)	15.80	5.20	13.30	1.48	Rejected (Fails C1)
Furfural	18.60	14.90	5.10	1.49	Rejected (Fails C1)

Table S1: continued

**Solvent**	δ_d (MPa$^{1/2}$)	δ_p (MPa$^{1/2}$)	δ_h (MPa$^{1/2}$)	RED	**Outcome & Reason**
Coumarin	20.00	12.50	6.70	1.54	Rejected (Fails C1)
Tetrahydrofurfuryl alcohol	17.80	8.20	12.90	1.56	Rejected (Fails C1)
Benzyl alcohol	18.40	6.30	13.70	1.66	Rejected (Fails C1)
m-Cresol	18.50	6.50	13.70	1.67	Rejected (Fails C1)
DMF	17.40	13.70	11.30	1.69	Rejected (Fails C1)
Acetonitrile	15.30	18.00	6.10	1.74	Rejected (Fails C1)
Sobrerol	16.67	6.46	16.08	1.84	Rejected (Fails C1)
2-propanol	15.80	6.10	16.40	1.87	Rejected (Fails C1)
DMSO	18.40	16.40	10.20	1.91	Rejected (Fails C1)
1-propanol	16.00	6.80	17.40	2.00	Rejected (Fails C1)
formic acid	14.30	11.90	16.60	2.12	Rejected (Fails C1)
4-Nitrophenol	20.00	14.50	14.20	2.24	Rejected (Fails C1)
Ethanol	15.80	8.80	19.40	2.29	Rejected (Fails C1)
Methanol	15.10	12.30	22.30	2.77	Rejected (Fails C1)
Water	15.50	16.00	42.30	5.29	Rejected (Fails C1)

Solubility measurement data

Table S2: LDPE-1 Solubility Data

Solvent	T/K	w_{PE}
Toluene	338.5	0.0193
Toluene	340.6	0.0405
Toluene	340.0	0.0451
Toluene	342.2	0.0629
Toluene	342.5	0.0701
Toluene	344.2	0.1015
Toluene	345.8	0.1218
Toluene	347.0	0.1435

Table S2: continued

Solvent	T/K	w_{PE}
Toluene	348.4	0.1684
Toluene	350.2	0.2080
Toluene	351.4	0.2242
Toluene	352.3	0.2670
n-Dodecane	351.3	0.0193
n-Dodecane	351.2	0.0203
n-Dodecane	356.0	0.0463
n-Dodecane	356.8	0.0524
n-Dodecane	357.0	0.0770
n-Dodecane	358.4	0.0802
n-Dodecane	360.4	0.1224
n-Dodecane	361.0	0.1254
n-Dodecane	360.8	0.1559
n-Dodecane	360.8	0.1691
n-Dodecane	363.2	0.2055
n-Dodecane	362.4	0.2152
n-Dodecane	363.8	0.2471
n-Dodecane	363.2	0.2522
n-Dodecane	364.4	0.2867
n-Dodecane	364.5	0.2938
Decalin	338.2	0.0193
Decalin	340.6	0.0405
Decalin	342.2	0.0629
Decalin	344.4	0.0848
Decalin	343.5	0.1000
Decalin	345.2	0.1243
Decalin	348.0	0.1538
Decalin	350.0	0.1940
Decalin	350.0	0.2157
Decalin	352.3	0.2515

Table S2: continued

Solvent	T/K	w_{PE}
p-Xylene	336.2	0.0174
p-Xylene	340.2	0.0311
p-Xylene	344.8	0.0491
p-Xylene	345.0	0.0816
p-Xylene	347.2	0.1003
p-Xylene	347.8	0.1217
p-Xylene	347.8	0.1449
p-Xylene	348.8	0.1580
p-Xylene	349.5	0.1887
p-Xylene	350.0	0.2097
p-Xylene	351.2	0.2367
p-Xylene	351.2	0.2475
p-Xylene	353.8	0.2735
p-Xylene	353.4	0.2912
p-Xylene	353.6	0.3083
p-Xylene	354.3	0.3279
Mesitylene	338.0	0.0126
Mesitylene	342.2	0.0379
Mesitylene	346.0	0.0648
Mesitylene	347.8	0.0894
Mesitylene	348.0	0.1155
Mesitylene	350.3	0.1383
Mesitylene	352.0	0.1609
Mesitylene	351.8	0.1737
Mesitylene	351.8	0.1964
Mesitylene	352.8	0.2149
Mesitylene	353.2	0.2358
Mesitylene	353.8	0.2554
Mesitylene	355.8	0.2829
Mesitylene	355.2	0.2939

Table S2: continued

Solvent	T/K	w_{PE}
Mesitylene	356.2	0.3075
Mesitylene	356.8	0.3198
Limonene	339.0	0.0085
Limonene	343.6	0.0292
Limonene	349.4	0.0802
Limonene	351.2	0.1162
Limonene	352.2	0.1778
Limonene	354.2	0.1998
Limonene	355.8	0.2377
Limonene	357.0	0.2655
p-Cymene	340.5	0.0075
p-Cymene	346.2	0.0213
p-Cymene	347.0	0.0271
p-Cymene	347.2	0.0385
p-Cymene	349.5	0.0571
p-Cymene	350.8	0.0856
p-Cymene	351.8	0.1054
p-Cymene	353.6	0.1231
p-Cymene	352.5	0.1343
p-Cymene	354.8	0.1555
p-Cymene	354.0	0.1794
p-Cymene	354.3	0.2024
p-Cymene	356.3	0.2279
p-Cymene	357.2	0.2441
p-Cymene	357.8	0.2635
p-Cymene	358.6	0.2808
Dibutoxymethane	355.6	0.0114
Dibutoxymethane	357.4	0.0200
Dibutoxymethane	359.2	0.0339
Dibutoxymethane	361.0	0.0564

Table S2: continued

Solvent	T/K	w_{PE}
Dibutoxymethane	361.0	0.0665
Dibutoxymethane	362.3	0.0881
Dibutoxymethane	362.5	0.1020
Dibutoxymethane	363.6	0.1217
Dibutoxymethane	364.0	0.1510
Dibutoxymethane	363.5	0.1726
Dibutoxymethane	364.8	0.2029
Dibutoxymethane	365.3	0.2269
Dibutoxymethane	366.8	0.2609
Cyclohexanone	360.2	0.0081
Cyclohexanone	363.3	0.0190
Cyclohexanone	364.2	0.0308
Cyclohexanone	365.0	0.0393
Cyclohexanone	366.2	0.0582
Cyclohexanone	367.0	0.0658
Cyclohexanone	366.8	0.0755
Cyclohexanone	367.8	0.1087
Cyclohexanone	367.3	0.1244
Cyclohexanone	367.3	0.1306
Cyclohexanone	368.0	0.1511
Cyclohexanone	368.6	0.1683
Cyclohexanone	368.0	0.1813
Cyclohexanone	367.8	0.2006
Cyclohexanone	367.2	0.2259
Cyclohexanone	368.8	0.2484
Cyclohexanone	368.8	0.2484
Cyclohexanone	367.3	0.2744
α-Pinene	338.5	0.0134
α-Pinene	343.8	0.0367
α-Pinene	347.2	0.0863

Table S2: continued

Solvent	T/K	w_{PE}
α-Pinene	350.8	0.1390
α-Pinene	353.0	0.1910
α-Pinene	355.8	0.2531
α-Pinene	358.0	0.2927
α-Pinene	360.5	0.3497

Table S3: LDPE-2 Solubility Data

Solvent	T/K	w_{PE}
Toluene	347.8	0.0074
Toluene	348.7	0.0100
Toluene	348.0	0.0219
Toluene	348.8	0.0231
Toluene	348.6	0.0319
Toluene	349.7	0.0451
Toluene	349.4	0.0462
Toluene	350.7	0.0664
Toluene	349.5	0.0672
Toluene	350.4	0.0868
Toluene	351.2	0.0892
Toluene	353.6	0.1101
Toluene	352.8	0.1132
Toluene	353.3	0.1280
Toluene	353.1	0.1321
Toluene	353.0	0.1495
Toluene	354.0	0.1639
Toluene	355.0	0.1889
Toluene	357.2	0.2113
Toluene	357.8	0.2295
Toluene	358.0	0.2586

Table S3: continued

Solvent	T/K	w_{PE}
Toluene	359.3	0.2780
n-Dodecane	358.2	0.0055
n-Dodecane	358.4	0.0117
n-Dodecane	359.5	0.0171
n-Dodecane	360.0	0.0183
n-Dodecane	363.0	0.0273
n-Dodecane	360.1	0.0412
n-Dodecane	363.2	0.0576
n-Dodecane	362.1	0.0613
n-Dodecane	363.8	0.0892
n-Dodecane	365.1	0.0893
n-Dodecane	364.7	0.1067
n-Dodecane	364.0	0.1158
n-Dodecane	365.4	0.1359
n-Dodecane	365.5	0.1386
n-Dodecane	367.8	0.1598
n-Dodecane	366.2	0.1625
n-Dodecane	368.0	0.1983
n-Dodecane	367.8	0.2259
n-Dodecane	368.5	0.2659
n-Dodecane	372.8	0.2974
Decalin	338.3	0.0060
Decalin	340.9	0.0072
Decalin	340.6	0.0134
Decalin	341.3	0.0135
Decalin	341.6	0.0224
Decalin	341.5	0.0308
Decalin	342.5	0.0469
Decalin	343.0	0.0521
Decalin	347.5	0.0688

Table S3: continued

Solvent	T/K	w_{PE}
Decalin	347.5	0.0743
Decalin	347.4	0.0973
Decalin	347.4	0.1068
Decalin	348.2	0.1209
Decalin	350.2	0.1320
Decalin	348.3	0.1401
Decalin	351.8	0.1509
Decalin	353.4	0.1830
Decalin	353.3	0.1964
Decalin	353.2	0.2165
Decalin	356.8	0.2414
Decalin	357.8	0.2650
Decalin	358.3	0.2753
p-Xylene	348.2	0.0129
p-Xylene	348.8	0.0139
p-Xylene	349.8	0.0400
p-Xylene	352.2	0.0688
p-Xylene	352.8	0.0697
p-Xylene	353.8	0.1079
p-Xylene	353.2	0.1134
p-Xylene	356.0	0.1720
p-Xylene	355.3	0.1738
p-Xylene	357.8	0.2069
p-Xylene	357.3	0.2109
p-Xylene	358.4	0.2515
p-Xylene	358.2	0.2525
p-Xylene	361.8	0.2893
p-Xylene	361.2	0.2994
Mesitylene	350.8	0.0123
Mesitylene	352.0	0.0356

Table S3: continued

Solvent	T/K	w_{PE}
Mesitylene	352.8	0.0435
Mesitylene	353.8	0.0662
Mesitylene	354.6	0.0757
Mesitylene	355.2	0.1047
Mesitylene	355.8	0.1172
Mesitylene	357.0	0.1532
Mesitylene	357.2	0.1713
Mesitylene	358.8	0.2096
Mesitylene	359.8	0.2472
Mesitylene	359.8	0.2707
Mesitylene	361.2	0.2823
Mesitylene	361.3	0.2852
Limonene	350.6	0.0080
Limonene	352.8	0.0299
Limonene	355.2	0.0828
Limonene	356.2	0.1169
Limonene	359.0	0.1577
Limonene	358.8	0.1822
Limonene	360.4	0.2087
Limonene	360.6	0.2261
Limonene	361.3	0.2419
Limonene	363.0	0.2762
Limonene	363.0	0.2827
Limonene	364.5	0.2858
p-Cymene	354.2	0.0132
p-Cymene	355.6	0.0347
p-Cymene	356.2	0.0556
p-Cymene	357.6	0.0750
p-Cymene	358.0	0.0786
p-Cymene	357.2	0.0913

Table S3: continued

Solvent	T/K	w_{PE}
p-Cymene	359.2	0.1177
p-Cymene	359.3	0.1312
p-Cymene	360.0	0.1327
p-Cymene	359.0	0.1464
p-Cymene	360.0	0.1661
p-Cymene	360.0	0.1827
p-Cymene	360.2	0.2014
p-Cymene	359.8	0.2265
p-Cymene	361.8	0.2318
p-Cymene	361.3	0.2494
p-Cymene	362.8	0.2707
p-Cymene	362.2	0.2764
p-Cymene	365.0	0.2852
Dibutoxymethane	364.2	0.0081
Dibutoxymethane	364.3	0.0096
Dibutoxymethane	365.2	0.0227
Dibutoxymethane	366.3	0.0291
Dibutoxymethane	366.5	0.0381
Dibutoxymethane	367.5	0.0481
Dibutoxymethane	368.2	0.0584
Dibutoxymethane	368.4	0.0724
Dibutoxymethane	368.6	0.0756
Dibutoxymethane	368.5	0.0842
Dibutoxymethane	368.8	0.0923
Dibutoxymethane	369.4	0.1084
Dibutoxymethane	369.2	0.1140
Dibutoxymethane	369.8	0.1403
Dibutoxymethane	369.3	0.1530
α-Pinene	347.6	0.0110
α-Pinene	349.6	0.0355

Table S3: continued

Solvent	T/K	w_{PE}
α-Pinene	353.8	0.0891
α-Pinene	356.2	0.1358
α-Pinene	358.2	0.1940
α-Pinene	363.0	0.2560
α-Pinene	363.8	0.2913
α-Pinene	368.0	0.3529

Table S4: LDPE-3 Solubility Data

Solvent	T/K	w_{PE}
Toluene	366.0	0.0069
Toluene	366.0	0.0177
Toluene	366.8	0.0524
Toluene	367.2	0.0905
Toluene	369.2	0.1137
Toluene	369.2	0.1329
Toluene	370.8	0.1435
Toluene	372.0	0.1895
Toluene	374.3	0.2526
n-Dodecane	379.0	0.0322
n-Dodecane	380.8	0.0885
n-Dodecane	382.6	0.1410
n-Dodecane	384.6	0.1980
n-Dodecane	384.5	0.2335
n-Dodecane	386.2	0.2976
n-Dodecane	388.6	0.3530
Decalin	363.3	0.0058
Decalin	365.0	0.0343
Decalin	367.8	0.0908
Decalin	370.0	0.1369

Table S4: continued

Solvent	T/K	w_{PE}
Decalin	371.8	0.1905
Decalin	373.6	0.2453
Decalin	375.8	0.2932
Decalin	378.4	0.3444
p-Xylene	365.8	0.0096
p-Xylene	367.8	0.0351
p-Xylene	369.2	0.0873
p-Xylene	371.2	0.1369
p-Xylene	373.6	0.1883
p-Xylene	375.2	0.2529
p-Xylene	376.8	0.2955
p-Xylene	381.8	0.3512
Mesitylene	367.4	0.0114
Mesitylene	368.8	0.0337
Mesitylene	370.8	0.0890
Mesitylene	373.2	0.1379
Mesitylene	374.5	0.1848
Mesitylene	376.5	0.2491
Mesitylene	378.0	0.2888
Mesitylene	381.0	0.3501
Limonene	372.3	0.0124
Limonene	372.8	0.0361
Limonene	375.0	0.0887
Limonene	377.2	0.1385
Limonene	378.2	0.1932
Limonene	381.5	0.2536
Limonene	381.0	0.2927
Limonene	383.4	0.3466
p-Cymene	372.8	0.0128
p-Cymene	373.8	0.0341

Table S4: continued

Solvent	T/K	w_{PE}
p-Cymene	375.3	0.0913
p-Cymene	377.4	0.1366
p-Cymene	378.3	0.1904
p-Cymene	380.0	0.2544
p-Cymene	380.8	0.2916
p-Cymene	383.0	0.3542
Dibutoxymethane	381.0	0.0122
Dibutoxymethane	381.5	0.0345
Dibutoxymethane	382.5	0.0824
Dibutoxymethane	384.2	0.1293
Dibutoxymethane	385.6	0.1792
Dibutoxymethane	385.8	0.2346
Dibutoxymethane	386.8	0.2767
Dibutoxymethane	389.5	0.3285
Cyclohexanone	386.8	0.0108
Cyclohexanone	387.8	0.0338
Cyclohexanone	387.8	0.0793
Cyclohexanone	388.3	0.1767
Cyclohexanone	388.2	0.2389
Cyclohexanone	388.2	0.2769
Cyclohexanone	389.4	0.3311
α-Pinene	368.0	0.0125
α-Pinene	368.8	0.0343
α-Pinene	371.2	0.0859
α-Pinene	374.6	0.1337
α-Pinene	376.4	0.1878
α-Pinene	378.0	0.2546
α-Pinene	379.2	0.2937
α-Pinene	383.8	0.3491

Table S5: MDPE Solubility Data

Solvent	T/K	w_{PE}
Toluene	343.6	0.0196
Toluene	348.4	0.0494
Toluene	350.8	0.0675
Toluene	350.8	0.0728
Toluene	351.5	0.1102
Toluene	353.8	0.1339
Toluene	353.8	0.1561
Toluene	354.5	0.1681
Toluene	355.5	0.1963
Toluene	357.2	0.2283
Toluene	358.0	0.2611
n-Dodecane	358.8	0.0177
n-Dodecane	358.3	0.0217
n-Dodecane	361.8	0.0446
n-Dodecane	363.2	0.0504
n-Dodecane	364.4	0.0521
n-Dodecane	364.4	0.0772
n-Dodecane	366.0	0.1234
n-Dodecane	366.8	0.1292
n-Dodecane	366.6	0.1640
n-Dodecane	366.5	0.1645
n-Dodecane	368.6	0.2130
n-Dodecane	368.4	0.2151
n-Dodecane	368.5	0.2446
n-Dodecane	368.5	0.2493
n-Dodecane	370.6	0.2929
n-Dodecane	370.8	0.2971
Decalin	344.0	0.0178
Decalin	346.8	0.0467

Table S5: continued

Solvent	T/K	w_{PE}
Decalin	348.6	0.0662
Decalin	350.5	0.1064
Decalin	351.6	0.1426
Decalin	354.0	0.1804
Decalin	354.4	0.1894
Decalin	355.5	0.1963
Decalin	355.8	0.2117
Decalin	357.2	0.2283
Decalin	357.3	0.2478
Decalin	357.2	0.2511
p-Xylene	346.0	0.0134
p-Xylene	347.8	0.0178
p-Xylene	349.2	0.0407
p-Xylene	351.5	0.0727
p-Xylene	352.3	0.0786
p-Xylene	354.0	0.1117
p-Xylene	354.6	0.1170
p-Xylene	355.8	0.1631
p-Xylene	355.0	0.1679
p-Xylene	357.2	0.2078
p-Xylene	358.0	0.2150
p-Xylene	358.2	0.2387
p-Xylene	359.0	0.2470
p-Xylene	361.3	0.2962
p-Xylene	360.2	0.2977
Mesitylene	347.8	0.0141
Mesitylene	350.0	0.0327
Mesitylene	351.0	0.0396
Mesitylene	352.3	0.0658
Mesitylene	353.2	0.0723

Table S5: continued

Solvent	T/K	w_{PE}
Mesitylene	355.8	0.1103
Mesitylene	355.0	0.1154
Mesitylene	357.2	0.1560
Mesitylene	358.0	0.2118
Mesitylene	359.3	0.2217
Mesitylene	360.0	0.2333
Mesitylene	359.8	0.2513
Mesitylene	361.2	0.2732
Mesitylene	362.3	0.2889
Mesitylene	362.2	0.2889
Limonene	348.3	0.0094
Limonene	353.0	0.0351
Limonene	353.8	0.0584
Limonene	354.5	0.0789
Limonene	356.8	0.1189
Limonene	359.0	0.1574
Limonene	358.2	0.1766
Limonene	359.6	0.2089
Limonene	360.5	0.2285
Limonene	361.2	0.2556
Limonene	364.0	0.2827
Limonene	364.2	0.2981
p-Cymene	350.2	0.0088
p-Cymene	354.3	0.0292
p-Cymene	355.6	0.0757
p-Cymene	357.2	0.0852
p-Cymene	359.0	0.1092
p-Cymene	359.8	0.1229
p-Cymene	359.6	0.1537
p-Cymene	360.2	0.1675

Table S5: continued

Solvent	T/K	w_{PE}
p-Cymene	360.0	0.1698
p-Cymene	360.0	0.1835
p-Cymene	361.8	0.2009
p-Cymene	362.6	0.2223
p-Cymene	361.8	0.2259
p-Cymene	361.6	0.2436
p-Cymene	362.4	0.2581
p-Cymene	363.3	0.2708
p-Cymene	365.2	0.2889
Dibutoxymethane	361.2	0.0049
Dibutoxymethane	360.3	0.0090
Dibutoxymethane	363.2	0.0149
Dibutoxymethane	365.0	0.0248
Dibutoxymethane	364.8	0.0317
Dibutoxymethane	366.2	0.0480
Dibutoxymethane	367.0	0.0559
Dibutoxymethane	367.2	0.0721
Dibutoxymethane	368.2	0.0916
Dibutoxymethane	368.4	0.1089
Dibutoxymethane	369.2	0.1109
Dibutoxymethane	369.8	0.1322
Dibutoxymethane	369.6	0.1418
Dibutoxymethane	369.8	0.1506
Cyclohexanone	369.5	0.0114
Cyclohexanone	371.3	0.0268
Cyclohexanone	372.2	0.0696
Cyclohexanone	372.4	0.0924
Cyclohexanone	372.4	0.1313
Cyclohexanone	372.5	0.1546
Cyclohexanone	372.8	0.1697

Table S5: continued

Solvent	T/K	w_{PE}
Cyclohexanone	373.0	0.2231
Cyclohexanone	373.2	0.2579
α-Pinene	347.5	0.0113
α-Pinene	352.0	0.0354
α-Pinene	355.3	0.0881
α-Pinene	357.2	0.1374
α-Pinene	359.0	0.1892
α-Pinene	360.8	0.2535
α-Pinene	362.8	0.2935
α-Pinene	365.2	0.3503

Parameter tables

Table S6: Unlike group parameters (excluding association) used with the SAFT-γ Mie approach. CR indicates parameters were calculated through combining rules.[1]

k	l	group k	group l	(ε_{kl}/k_B) / K	λ^r_{kl}	Ref.
1	1	CH_3	CH_3	256.77	15.050	1
1	2	CH_3	CH_2	350.77	CR	1
1	3	CH_3	CH	387.48	CR	2
1	4	CH_3	$aCCH_3$	358.58	CR	3
1	5	CH_3	aCH	305.81	CR	2
1	6	CH_3	aCCH	455.85	CR	2
1	7	CH_3	cCH_2	355.95	CR	2
1	8	CH_3	cCH	690.17	CR	4
1	9	CH_3	mO	261.63	CR	5
1	10	CH_3	cCO	264.18	CR	6
2	2	CH_2	CH_2	473.39	19.871	1
2	3	CH_2	CH	506.21	CR	2
2	4	CH_2	$aCCH_3$	569.18	CR	3

Table S6: continued

k	l	group k	group l	(ε_{kl}/k_B) / K	λ^r_{kl}	Ref.
2	5	CH_2	aCH	415.64	CR	2
2	6	CH_2	aCCH	345.8	CR	2
2	7	CH_2	cCH_2	469.67	CR	2
2	8	CH_2	cCH	522.57	CR	4
2	9	CH_2	mO	353.78	CR	5
2	10	CH_2	cCO	368.09	CR	6
3	3	CH	CH	95.621	8.0000	2
3	4	CH	$aCCH_3$	769.36	8.0000	7
3	5	CH	aCH	441.43	CR	2
3	6	CH	aCCH	67.51	CR	2
3	7	CH	cCH_2	570.45	CR	2
3	8	CH	cCH	CR	CR	—
3	9	CH	mO	CR	CR	—
3	10	CH	cCO	196.23	CR	—
4	4	$aCCH_3$	$aCCH_3$	651.41	23.627	3,8
4	5	$aCCH_3$	aCH	471.23	CR	8
4	6	$aCCH_3$	aCCH	CR	CR	—
4	7	$aCCH_3$	cCH_2	540.63	21.082	7
4	8	$aCCH_3$	cCH	792.29	CR	7
4	9	$aCCH_3$	mO	CR	CR	—
4	10	$aCCH_3$	cCO	CR	CR	—
5	5	aCH	aCH	371.53	14.756	2
5	6	aCH	aCCH	429.16	CR	2
5	7	aCH	cCH_2	393.05	15.377	7
5	8	aCH	cCH	377.21	CR	7
5	9	aCH	mO	CR	CR	—
5	10	aCH	cCO	392.49	CR	—
6	6	aCCH	aCCH	61.325	8.0000	2
6	7	aCCH	cCH_2	CR	CR	—
6	8	aCCH	cCH	CR	CR	—
6	9	aCCH	mO	CR	CR	—

Table S6: continued

k	l	group k	group l	(ε_{kl}/k_B) / K	λ^r_{kl}	Ref.
6	10	aCCH	cCO	CR	CR	—
7	7	cCH_2	cCH_2	477.36	20.386	—
7	8	cCH_2	cCH	321.71	CR	4
7	9	cCH_2	mO	CR	CR	—
7	10	cCH_2	cCO	383.29	14.939	—
8	8	cCH	cCH	699.92	8.0000	4
8	9	cCH	mO	CR	CR	—
8	10	cCH	cCO	250.81	CR	—
9	9	mO	mO	248.2	10.127	5
9	10	mO	cCO	CR	CR	—
10	10	cCO	cCO	477.98	8.0	—

Influence of molecular weight on PE solubility predictions

To quantify the molecular weight at which polymer solubility reaches a plateau for each isotherm, a numerical procedure based on finite differences is applied. For each isotherm, the data were first sorted in ascending order of polymer molecular weight M_w, and the solubility variable w_{PE} was analysed as a function of M_w.

The first-order forward finite difference was used to estimate the slope between consecutive data points:

$$\left(\frac{\Delta w}{\Delta M_w}\right)_i = \frac{w_{i+1} - w_i}{M_{w,i+1} - M_{w,i}} \tag{1}$$

To identify the onset of the plateau region, we defined a threshold slope of $\varepsilon = 10^{-6}$ and required that this condition be satisfied over a window of ten consecutive points. That is, a plateau was said to begin at the first M_w where:

$$\left|\frac{\Delta w}{\Delta M_w}\right|_i, \quad \left|\frac{\Delta w}{\Delta M_w}\right|_{i+1}, \quad \left|\frac{\Delta w}{\Delta M_w}\right|_{i+2}, \quad \ldots, \quad \left|\frac{\Delta w}{\Delta M_w}\right|_{i+9} < \varepsilon \tag{2}$$

This criterion reduces sensitivity to local noise and ensures a sustained plateauing of the solubility curve. The corresponding molecular weight at the end of the qualifying window (i.e., $M_{w,i+10}$) was recorded as the onset of the plateau.

This procedure was applied independently to each isotherm. The average plateau onset molecular weight was then computed across all temperatures for which a plateau was detected.

In Figure S1, we analyse the impact of the polymer molecular weight on the solubility predictions using $T^{\text{fus}}_{\text{LDPE-2}}$=389.15 K and $\Delta h^{\text{fus}}_{\text{LDPE-2}}$=293.0 J/g for PE+toluene and decalin at seven selected temperatures. The M_w of PE takes 1000 values between 4 kg/mol and 270 kg/mol.

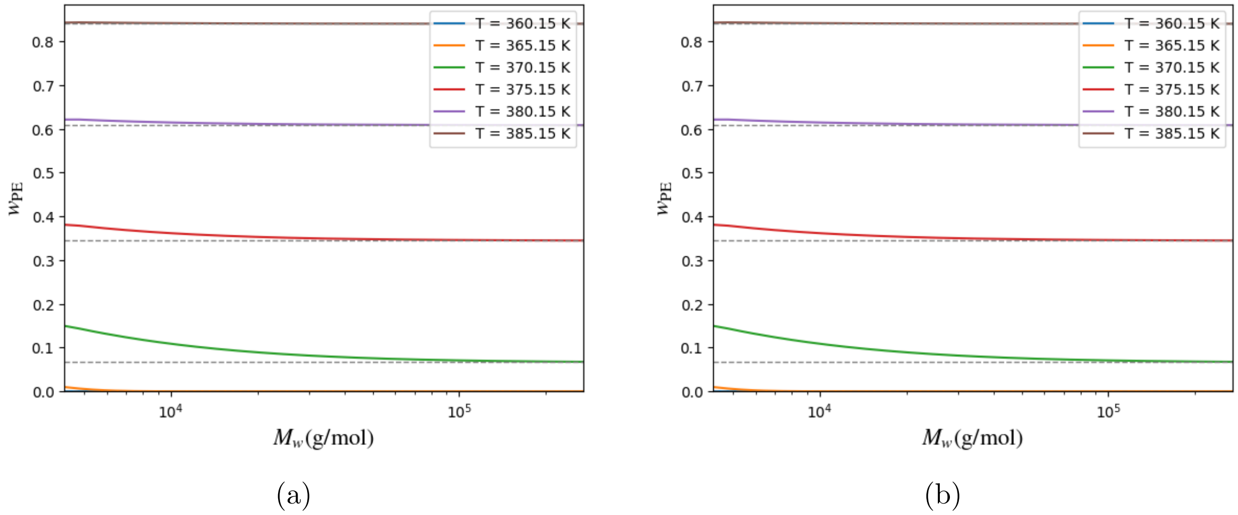

Figure S1: Impact of the molecular weight on the orthobaric solid-liquid equilibria at the solvent vapour pressure $P = P^{\text{vap}}_{\text{sol}}$ for PE + n-dodecane (a) and dibutoxymethane (b). $\Delta h^{\text{fus}}_{\text{LDPE-2}}$=293.0 J/g and $T^{\text{fus}}_{\text{LDPE-2}}$ = 389.15 K were used in the solubility calculations.

PE solubility predictions using molecular weights sampled from a Schulz-Flory distribution

The weight distribution function $W^{(0)}(m)$ for the Shulz–Flory model[9] is given by:

$$W^{(0)}(m) = \frac{b^{a+1}}{\Gamma(a+1)} m^a e^{-bm}, \tag{3}$$

where a and b are distribution parameters, m is the chain length, and Γ is the gamma function.

To normalize this distribution, the following gamma integral identity is used:

$$\int_0^\infty dm\, m^{a-1} e^{-bm} = b^{-a}\Gamma(a). \tag{4}$$

The number-average chain length $\langle m \rangle$ is:

$$\langle m \rangle = \frac{a}{b}. \tag{5}$$

The polydispersity index I_p, defined as the ratio of weight-average (M_w) to number-average (M_n) molecular weight, is:

$$I_p = \frac{a+1}{a}. \tag{6}$$

We compute the Schulz-Flory distribution of the LDPE-2 sample for which M_w=104952 g/mol and M_n=15975 g/mol. This corresponds to an I_p=6.57 and

$$\langle m \rangle = \frac{M_n - 2M_{\mathrm{w,CH_3}}}{M_{\mathrm{w,CH_2}}} = 1138$$

In Figure S3, we analyse the impact of the polymer molecular weight on the orthobaric solid-liquid equilibria predictions using $T^{\mathrm{fus}}_{\text{LDPE-2}}$=389.15 K and $\Delta h^{\mathrm{fus}}_{\text{LDPE-2}}$=293.0 J/g for PE+toluene.We carry out calculations using the M_w of PE 1000 sampled from the Schulz-Flory distribution

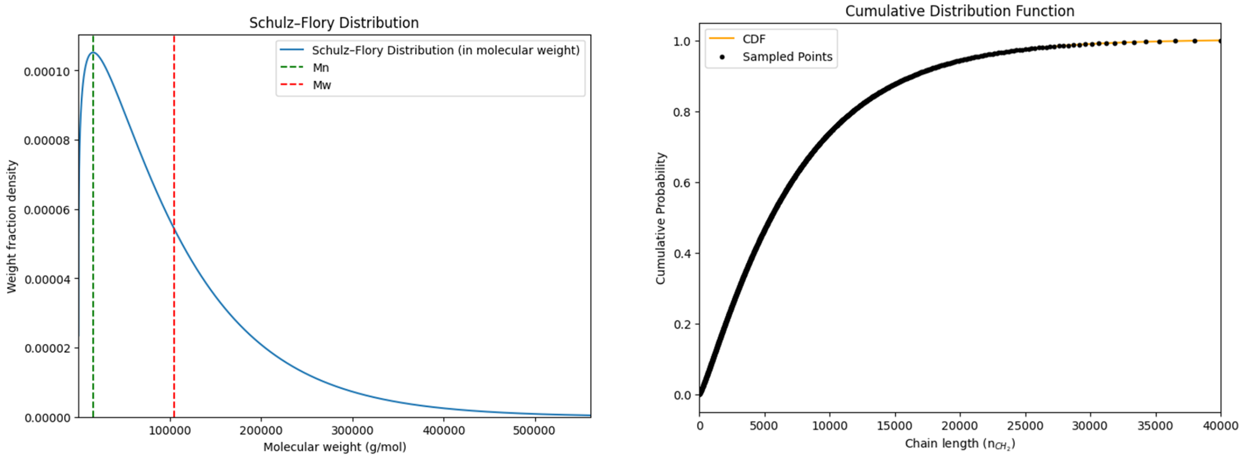

(a) (b)

Figure S2: Molecular weight distribution and corresponding CDF of distribution of the LDPE-2 sample, modeled with a Shulz-Flory distribution.[9]

with equally spaced probabilities. We observe that the solid-liquid equilibria curves overlap for $M_w > 40$ kg/mol. According to our model, this may indicate that all PE chains will have transitioned to the liquid phase at temperatures above the solid-liquid equilibrium temperature predicted when using $M_w > 40$ kg/mol.

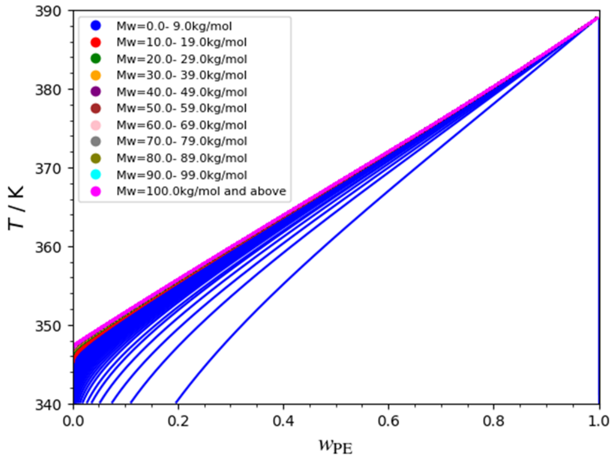

Figure S3: Orthobaric solid-liquid equilibria at the solvent vapour pressure $P = P_{\mathrm{sol}}^{\mathrm{vap}}$ of 1000 PEs sampled according to the Schulz-Flory distribution + toluene. $\Delta h_{\mathrm{LDPE\text{-}2}}^{\mathrm{fus}}$=293.0 J/g and $T_{\mathrm{LDPE\text{-}2}}^{\mathrm{fus}}$=389.15 K are used.

Modelling of selected PE + solvent mixtures with SAFT-γ Mie

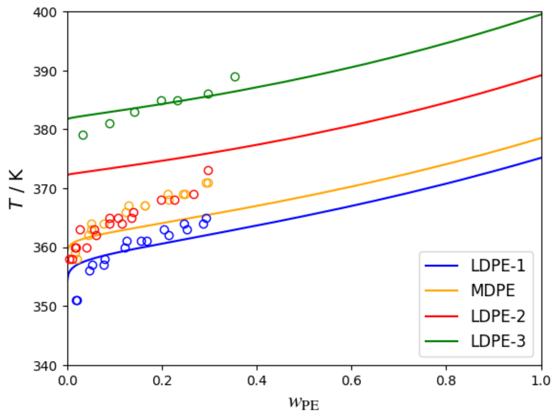

Figure S4: Orthobaric SLE at the solvent vapour pressure $P = P_{\text{sol}}^{\text{vap}}$ of LDPE-1, MDPE, LDPE-2 and LDPE-3 in n-dodecane. $\Delta h^{\text{fus}}_{\text{LDPE-1,LDPE-2,MDPE,LDPE-3}}$=293.0 J/g, $T^{\text{fus}}_{\text{LDPE-1}}$=375.2 K, $T^{\text{fus}}_{\text{MDPE}}$=378.5 K, $T^{\text{fus}}_{\text{LDPE-2}}$=389.2 K and $T^{\text{fus}}_{\text{LDPE-3}}$=399.5 K. The circles represent experimental data from this work

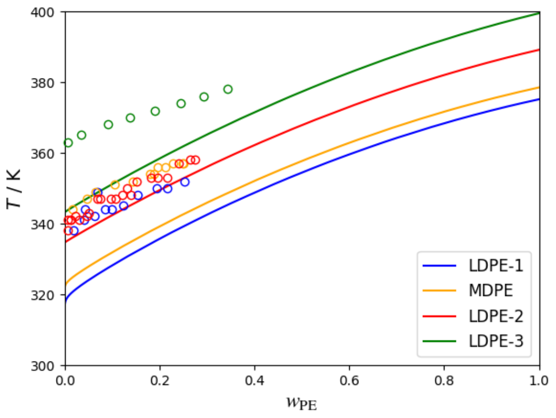

(a)

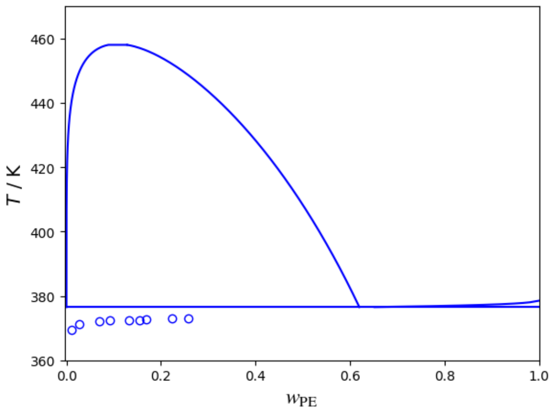

(b)

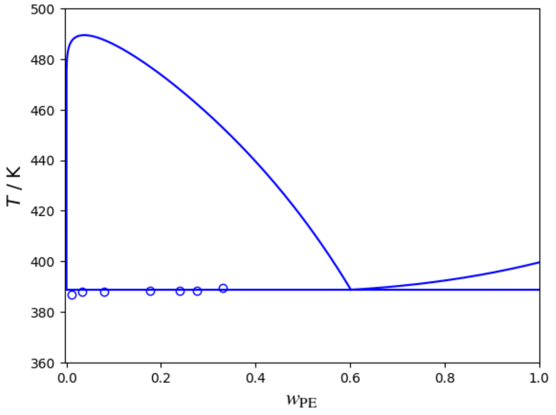

(c)

Figure S5: Phase diagrams of PE + cyclic solvents. (a) Orthobaric SLE at$P = P_{\mathrm{sol}}^{\mathrm{vap}}$ of LDPE-1, MDPE, LDPE-2 and LDPE-3 in decalin. $\Delta h^{\mathrm{fus}}_{\mathrm{LDPE\text{-}1,MDPE,LDPE\text{-}2,LDPE\text{-}3}}$=293.0 J/g, $T^{\mathrm{fus}}_{\mathrm{LDPE\text{-}1}}$=375.2 K, $T^{\mathrm{fus}}_{\mathrm{MDPE}}$=378.5 K, $T^{\mathrm{fus}}_{\mathrm{LDPE\text{-}2}}$=389.2 K and $T^{\mathrm{fus}}_{\mathrm{LDPE\text{-}3}}$=399.5 K. (b) Phase diagram of the mixtures of MDPE and LDPE-3 and cyclohexanone at $P = P_{\mathrm{sol}}^{\mathrm{vap}}$. The curves represent calculations with SAFT-γ Mie. The circles represent experimental data from this work.

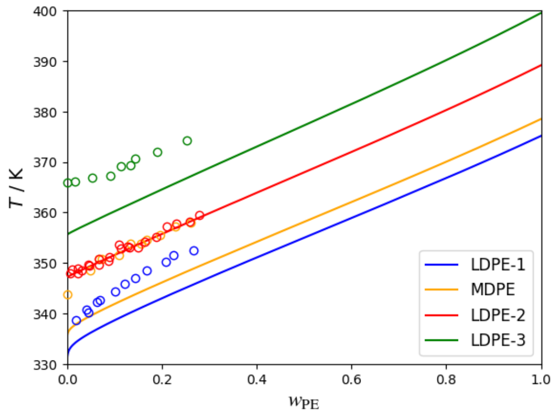

(a)

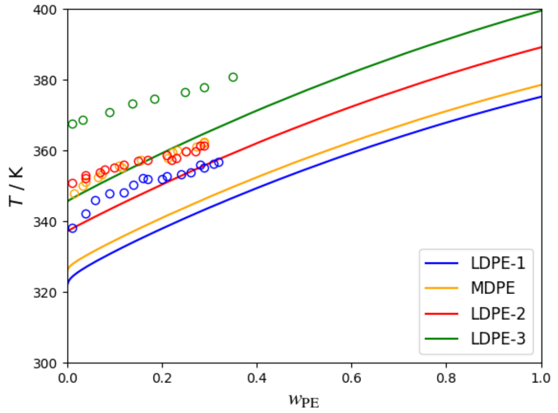

(b)

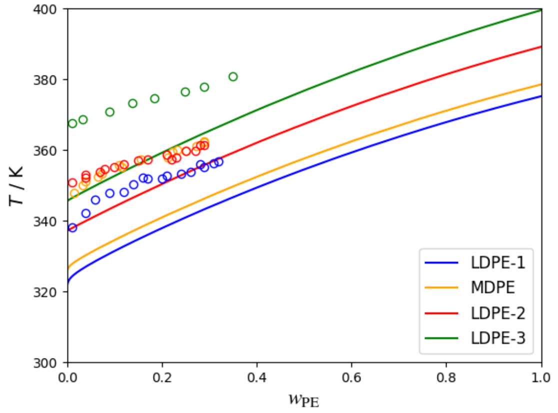

(c)

Figure S6: Orthobaric SLE at the solvent vapour pressure $P = P_{\text{sol}}^{\text{vap}}$ of LDPE-1, MDPE, LDPE-2 and LDPE-3 in three aromatic solvents: (a) toluene, (b) *p*-Xylene and (c) mesitylene. $\Delta h^{\text{fus}}_{\text{LDPE-1,LDPE-2,MDPE,LDPE-3}}$=293.0 J/g, $T^{\text{fus}}_{\text{LDPE-1}}$=375.2 K, $T^{\text{fus}}_{\text{MDPE}}$=378.5 K, $T^{\text{fus}}_{\text{LDPE-2}}$=389.2 K and $T^{\text{fus}}_{\text{LDPE-3}}$=399.5 K. The curves represent calculations with SAFT-γ Mie. The circles represent experimental data from this work.

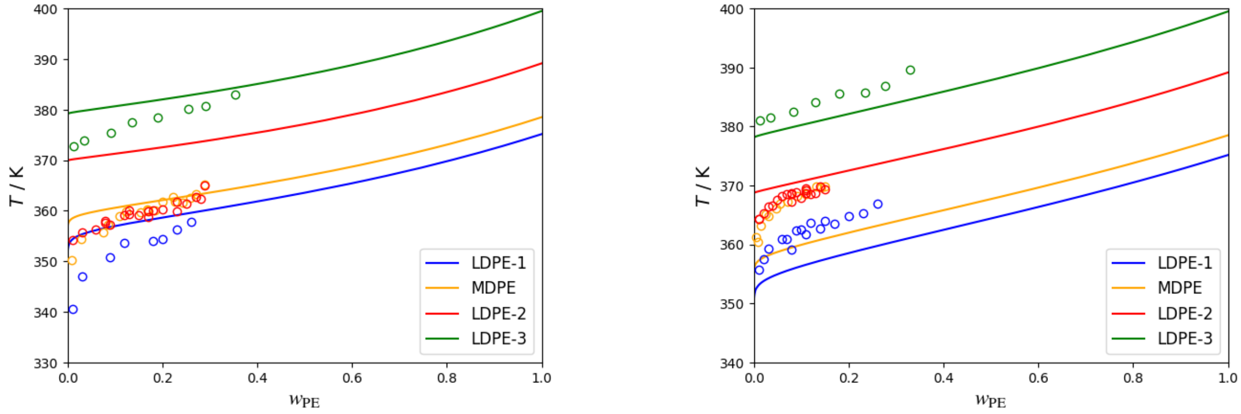

(a) (b)

Figure S7: Orthobaric SLE at the solvent vapour pressure $P = P_{\text{sol}}^{\text{vap}}$ of LDPE-1, MDPE, LDPE-2 and LDPE-3 in two green solvents: (a) *p*-cymene and (b) dibutoxymethane. $\Delta h^{\text{fus}}_{\text{LDPE-1,LDPE-2,MDPE,LDPE-3}}$=293.0 J/g, $T^{\text{fus}}_{\text{LDPE-1}}$=375.2 K, $T^{\text{fus}}_{\text{MDPE}}$=378.5 K, $T^{\text{fus}}_{\text{LDPE-2}}$=389.2 K and $T^{\text{fus}}_{\text{LDPE-3}}$=399.5 K. The curves represent calculations with SAFT-γ Mie. The circles represent experimental data from this work.

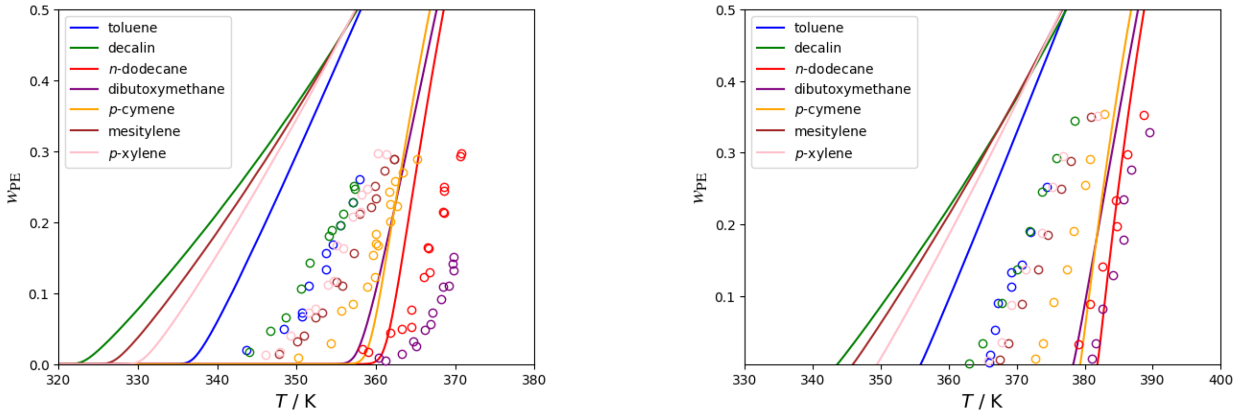

(a) (b)

Figure S8: Solubility of MDPE and LDPE-3 in toluene, decalin, *n*-dodecane, dibutoxymethane, *p*-cymene, mesitylene, *p*-xylene. The curves represent solubility calculations with SAFT-γ Mie. The circles represent experimental data from this work. $P = P_{\text{sol}}^{\text{vap}}$.

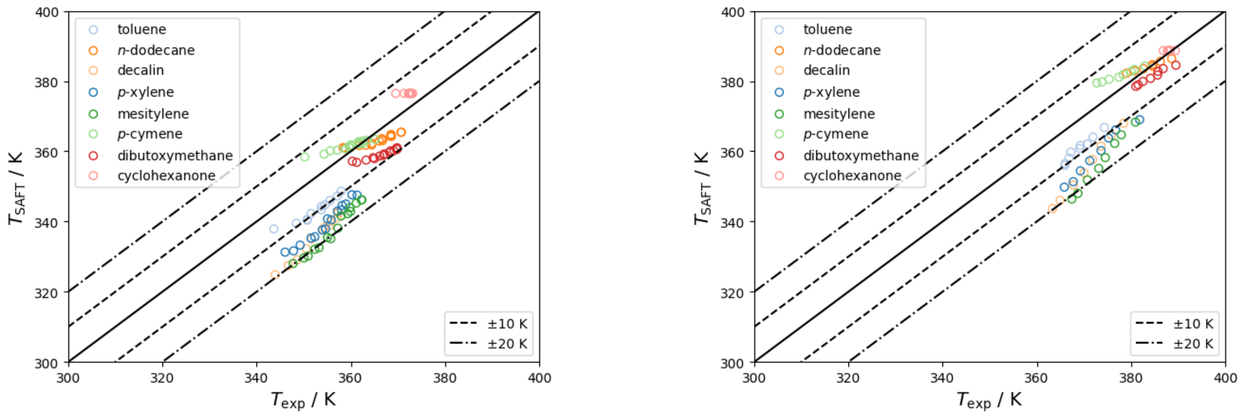

(a)

(b)

Figure S9: SLE temperature of (a) MDPE and (b) LDPE-3 calculated with SAFT-γ Mie vs experimental data from this work at the same w_{PE}. $P = P_{\mathrm{sol}}^{\mathrm{vap}}$.

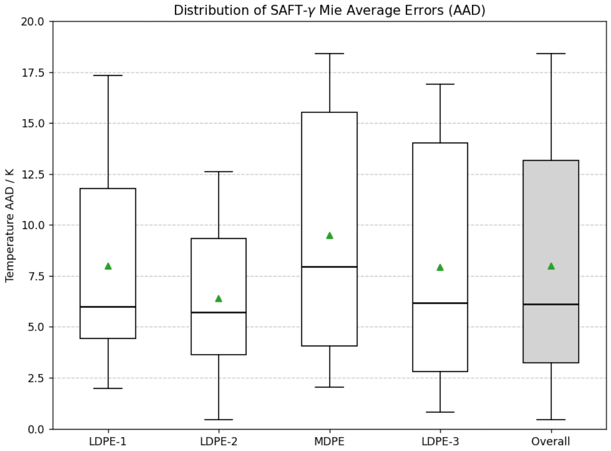

Figure S10: Box plot of the Absolute Average Deviations (AAD) for the different polymer samples. The data points used are the AAD values calculated for each solvent. The "Overall" box aggregates these AAD values across all four polymer samples to show the global performance distribution.

References

(1) Papaioannou, V.; Lafitte, T.; Avendaño, C.; Adjiman, C. S.; Jackson, G.; Müller, E. A.; Galindo, A. Group contribution methodology based on the statistical associating fluid theory for heteronuclear molecules formed from Mie segments. *The Journal of Chemical Physics* **2014**, *140*, 054107.

(2) Dufal, S.; Papaioannou, V.; Sadeqzadeh, M.; Pogiatzis, T.; Chremos, A.; Adjiman, C. S.; Jackson, G.; Galindo, A. Prediction of thermodynamic properties and phase behavior of fluids and mixtures with the SAFT-γ Mie group-contribution equation of state. *Journal of Chemical & Engineering Data* **2014**, *59*, 3272–3288.

(3) Hutacharoen, P.; Dufal, S.; Papaioannou, V.; Shanker, R. M.; Adjiman, C. S.; Jackson, G.; Galindo, A. Predicting the solvation of organic compounds in aqueous environments: from alkanes and alcohols to pharmaceuticals. *Industrial & Engineering Chemistry Research* **2017**, *56*, 10856–10876.

(4) Perdomo, F. A.; Khalit, S. H.; Adjiman, C. S.; Galindo, A.; Jackson, G. Description of the thermodynamic properties and fluid-phase behavior of aqueous solutions of linear, branched, and cyclic amines. *AIChE Journal* **2021**, *67*, e17194, _eprint: https://onlinelibrary.wiley.com/doi/pdf/10.1002/aic.17194.

(5) Burger, J.; Papaioannou, V.; Gopinath, S.; Jackson, G.; Galindo, A.; Adjiman, C. S. A hierarchical method to integrated solvent and process design of physical CO_2 absorption using the SAFT-γ Mie approach. *AIChE Journal* **2015**, *61*, 3249–3269.

(6) Paliwal, S.; Perdomo, F. A.; Bernet, T.; Alyazidi, A.; Haslam, A. J.; Jackson, G.; Galindo, A. Extending the SAFT-γ Mie group-contribution approach to model cyclic ketones: application to monoterpenoids. *Molecular Physics* **2025**, *0*, e2493320.

(7) Febra, S. A.; Bernet, T.; Mack, C.; McGinty, J.; Onyemelukwe, I. I.; Urwin, S. J.; Sefcik, J.; ter Horst, J. H.; Adjiman, C. S.; Jackson, G.; Galindo, A. Extending the SAFT-γ Mie approach to model benzoic acid, diphenylamine, and mefenamic acid: Solubility prediction and experimental measurement. *Fluid Phase Equilibria* **2021**, *540*, 113002.

(8) Papaioannou, V.; Calado, F.; Lafitte, T.; Dufal, S.; Sadeqzadeh, M.; Jackson, G.; Adjiman, C. S.; Galindo, A. Application of the SAFT-γ Mie group contribution equation of state to fluids of relevance to the oil and gas industry. *Fluid Phase Equilibria* **2016**, *416*, 104–119.

(9) Shultz, A. R.; Flory, P. J. Phase equilibria in polymer—solvent systems[1,2]. *Journal of the American Chemical Society* **1952**, *74*, 4760–4767, Publisher: American Chemical Society.
